# The Control Region of Mitochondrial DNA Shows an Unusual CpG and Non-CpG Methylation Pattern

**DOI:** 10.1093/dnares/dst029

**Published:** 2013-06-26

**Authors:** Dina Bellizzi, Patrizia D'Aquila, Teresa Scafone, Marco Giordano, Vincenzo Riso, Andrea Riccio, Giuseppe Passarino

**Affiliations:** 1 Department of Cell Biology, University of Calabria, Rende 87036, Italy; 2 Institute of Genetics and Biophysics—Adriano Buzzati Traverso, Napoli 80131, Italy

**Keywords:** mitochondrial D-loop region, 5-methylcytosine, 5-hydromethylcytosine, CpG methylation, non-CpG methylation

## Abstract

DNA methylation is a common epigenetic modification of the mammalian genome. Conflicting data regarding the possible presence of methylated cytosines within mitochondrial DNA (mtDNA) have been reported. To clarify this point, we analysed the methylation status of mtDNA control region (D-loop) on human and murine DNA samples from blood and cultured cells by bisulphite sequencing and methylated/hydroxymethylated DNA immunoprecipitation assays. We found methylated and hydroxymethylated cytosines in the L-strand of all samples analysed. MtDNA methylation particularly occurs within non-C-phosphate-G (non-CpG) nucleotides, mainly in the promoter region of the heavy strand and in conserved sequence blocks, suggesting its involvement in regulating mtDNA replication and/or transcription. We observed DNA methyltransferases within the mitochondria, but the inactivation of Dnmt1, Dnmt3a, and Dnmt3b in mouse embryonic stem (ES) cells results in a reduction of the CpG methylation, while the non-CpG methylation shows to be not affected. This suggests that D-loop epigenetic modification is only partially established by these enzymes. Our data show that DNA methylation occurs in the mtDNA control region of mammals, not only at symmetrical CpG dinucleotides, typical of nuclear genome, but in a peculiar non-CpG pattern previously reported for plants and fungi. The molecular mechanisms responsible for this pattern remain an open question.

## 1. Introduction

DNA methylation is the most studied epigenetic modification that occurs in all prokaryotic and eukaryotic organisms, with rare exception for yeast, roundworm, and fruit fly.^1^ In mammals, it is a post-replication modification in which a methyl group is covalently added to the 5-position of cytosines [5-methylcytosine (5mC)] that are part of symmetrical C-phosphate-G (CpG) dinucleotides. In plant genomes, DNA methylation can occur either symmetrically at cytosines in both CG and CHG (H = A, T, or C) contexts, or asymmetrically in a CHH context.^2^ On the contrary, non-CpG methylation in mammals is quite a rare event, although it has been recently described in embryonic stem cells,^3,4^ adult mouse brain,^5^ mouse germ cells,^6,7^ and in promoter regions of different genes.^8–10^ Both CpG and non-CpG methylation are determined and maintained by a family of conserved DNA methyltransferases (DNMTs).^11–14^ It has been widely demonstrated that CpG methylation status influences chromatin structure, thus regulating the accessibility of transcription factors to their DNA target sequences.^15–17^ Conversely, the biological significance of non-CpG methylation is currently unknown.

The detection of 5-hydroxymethylcytosine (5hmC) residues in different tissues^18,19^ and cells^20^ (mainly neurons, brain and embryonic stem cells) has recently been reported. 5hmC is generated through oxidation of 5mC by the TET family of methylcytosine dioxygenases.^21–23^ However, a role in 5hmC production was recently also ascribed to DNMT enzymes,^24^ suggesting that this species may not be exclusively considered an intermediate of the 5mC demethylation process, but also an important epigenetic marker regulating the pluripotency of stem cells, cellular development, aging, and carcinogenesis.^25^

Previous studies have reported the complete absence of 5mC in mitochondrial DNA (mtDNA) from *Paramecium aurelia*,^26^ *Xenopus leavis*,^27^ *Neurospora crassa*, and other species,^28^ mainly based on the identical restriction patterns obtained with the methyl-sensitive isoschizomers, *Hpa*II and *Msp*I. More recently, the lack of 5mC residues was confirmed by Maekawa *et al*. ^29^ by analysing cancer cell lines and tissues from patients with gastric and colorectal cancer. Conversely, different amounts of 5mC were observed in mtDNA of many other organisms, such as humans,^30^ mice,^31^ hamsters,^32^ and plants,^33^ although the distribution of the methylated cytosines has not been determined in any of these species. More recently, a mtDNA–protein interaction study suggested that this genome may be methylated and DNMTs artificially targeting the mitochondria have access to different sites on the mtDNA depending on the levels of protein occupancy.^34^ Finally, Shock *et al*.^35^ demonstrated an enrichment of mtDNA sequences by immunoprecipitation against 5mC and 5hmC and the translocation of DNA (cytosine-5-)-methyltransferase 1 (DNMT1) into the mitochondria.

The aim of this study was to investigate the presence of methylated residues of cytosines within mtDNA. In particular, the methylation status of the mtDNA control region (D-loop) was analysed both in blood DNA collected from human subjects and in DNA from cultured cells by bisulphite sequencing and, consecutively, by methylated/hydroxymethylated DNA immunoprecipitation assays. We focused on the above region because it is the control region of the mtDNA, it contains the main regulatory elements for its replication and expression and it is the most rapidly evolving region of this genome.^36^ The same analysis was also applied to DNA samples extracted from mouse blood and fibroblast cells. In addition, immunoblotting analyses were carried out to identify which of the three DNA (cytosine-5-)-methyltransferases (DNMT1, DNMT3A, and DNMT3B) and tet methylcytosine dioxygenases (TET1, TET2, and TET3) were located within the mitochondria. Finally, the potential role of the DNMTs in determining D-loop methylation status was investigated by applying the bisulphite sequencing procedure to DNA samples from wild-type (wt) and *Dnmt1^−/−^*, *Dnmt3a^−/−^*, and *Dnmt3b^−/−^* mouse embryonic stem (ES) cells.

## 2. Materials and Methods

### 2.1. Population sample

A total of 30 unrelated adult subjects (14 men and 16 women), 41–102 years old, participated to the present study. The Ethics committee of the University of Calabria approved the recruitment and the use of the information gathered, as well as the use of the biological specimens collected on 9th September 2004. All subjects lived in Calabria (South of Italy) and their origin in the area was ascertained up to the grandparents' generation. Health status was ascertained by medical visit and at that time peripheral blood samples were also obtained. Before the interview, each subject provided informed consent to permit her/his phenotypic and genetic data to be used anonymously for genetic studies.

### 2.2. Cell cultures

Human skin fibroblasts, HeLa, osteosarcoma 143B.TK^−^, and murine 3T3-L1 cells were grown in Dulbecco's Modified Eagle Medium (DMEM, Invitrogen) containing 4.5 g/l glucose and 110 µg/ml pyruvate, supplemented with 10% fetal bovine serum (FBS, Invitrogen) and 50 µg/ml gentamycin (Invitrogen). The Rho^0^ cell line, obtained by culturing 143B.TK^−^ in routine growth medium containing 50 ng/ml ethidium bromide with regular replenishment of medium for about 2 months, was maintained in DMEM supplemented with 10% FBS and 0.2 mM uridine (Sigma).

Wt and *Dnmt1^−/−^*, *Dnmt3a^−/−^*, and *Dnmt3b^−/−^* triple knock-out (TKO) mouse ES cells were grown in gelatinized culture dishes without feeder cells as reported by Tsumura *et al*.^37^

Cells were cultured in a water-humidified incubator at 37°C in 5% CO_2_/95% air.

### 2.3. DNA samples

Six millilitres of venous blood were drawn from each subject. Plasma/sera were used for routine laboratory analyses, while DNA was extracted from blood buffy coats following standard procedures.

DNA samples from human skin fibroblasts, HeLa, osteosarcoma 143B.TK^−^, Rho^0^, and murine 3T3-L1 cells were obtained by phenol/chloroform purification, while DNA samples from wt and *Dnmt1^−/−^*, *Dnmt3a^−/−^*, and *Dnmt3b^−/−^* (TKO) mouse ES cells^37^ were extracted by the Wizard Genomic DNA Purification Kit (Promega).

Mouse genomic DNA, isolated from whole blood of disease-free mice, was purchased from Promega Corporation.

### 2.4. Bisulphite treatment

Bisulphite conversion of each DNA sample was performed by using the EZ DNA Methylation-Direct Kit (Zymo Research), according to the manufacturer's protocol. Briefly, 1 µg of genomic DNA, previously incubated for 20 min at 50°C in proteinase K and purified by centrifugation, was added to 130 µl of CT Conversion Reagent in a final volume of 150 µl. The mix was incubated at 98°C for 10 min and, successively, at 64°C for 3.5 h. After adding 600 µl of M-Binding Buffer into a Zymo-Spin IC Column, each sample was loaded into the column and centrifuged at 16 000 *g* for 30 s. After adding 100 µl of M-Wash Buffer to the columns and a centrifugation at 16 000 *g* for 30 s, 200 µl of M-Desulphonation Buffer were added to the columns and incubated at room temperature (RT) for 20 min. Then, the solution was removed by centrifugation at 16 000 *g* for 30 s and the columns were washed twice with 200 µl of M-Wash Buffer. Deaminated DNA was eluted in 10 µl of M-Elution Buffer.

To ensure that cytosine conversion was complete, alternative bisulphite modifications were performed by using the EZ DNA Methylation-Gold Kit (Zymo Research), and the Qiagen's EpiTect Bisulfite Kit according to the manufacturer's protocol.

For each procedure of bisulphite treatment, unconverted primers randomly covering the entire mtDNA molecule, including those used for the D-loop analysis, were used in polymerase chain reaction (PCR) reactions, as negative controls.

### 2.5. Primer design for PCR reactions

Six and four sub-regions, covering the entire D-loop of humans (nt 16 024-576) and mice (nt 15 423–16 299), respectively, were isolated by PCR carried out on each bisulphite-converted DNA sample. In particular, as DNA strands are no longer complementary after sodium bisulphite treatment, we designed primers specifically amplifying the top (Light) and bottom (Heavy) strands of the bisulphite-converted DNA (Supplementary Tables S1 and S2). Moreover, some precautions were taken for primer design: (i) cytosines in forward primers and guanines in the reverse primers were replaced with thymines and adenines, respectively; (ii) cytosines within CpG sites were avoided; (iii) when possible, DNA regions characterized by low polymorphism content were preferred; (iv) short size of the amplicons was defined (range: 150–350 bp); (v) a 10-bp tag was added to the 5′- ends of some primers in order to increase the annealing temperature of the A–T enriched primer sequences. In addition, specificity for mtDNA target sequences of designed primers was tested on DNA extracted from osteosarcoma 143B.TK^−^ Rho^0^ cells, completely lacking of mtDNA.

### 2.6. Bisulphite sequencing

The PCR mixture (20 μl) contained 2 μl of bisulphite-treated DNA, Reaction Buffer 1×, TaqMaster PC Enhancer 1×, 0.5 μM of each primer, 0.2 mM dNTP mix (Promega), and 0.05 U of PCR enzyme (5′). The thermal profile used for the reaction included a 4-min heat activation of the enzyme at 95°C, followed by 45 cycles of denaturation at 94°C for 20 s, different annealing temperature (Supplementary Table S1) for 30 s, extension at 72°C for 1 min, then one cycle at 72°C for 3 min.

The obtained PCR products, previously purified by DNA Clean & Concentrator-5 Kit (Zymo Research), were cloned into pGEM-T Easy Vector (Promega) according to the manufacturer's protocol. In the set-up and validation of this procedure, 30 positive clones from five representative human DNA samples were analysed. Once determined the efficacy of the entire protocol, five clones for sample were analysed.

Therefore, plasmids were purified using ZR Plasmid Miniprep Classic (Zymo Research) and analysed by automated sequencing in a ABI PRISM 310 with the BigDye Terminator Cycle Sequencing Ready Reaction Kit (Applied Biosystems).

The effectiveness of the entire experimental procedure was also assayed by analysing: (i) CpGenome™ Universal Unmethylated DNA (Chemicon); (ii) unmethylated purified PCR products of each sub-region; and (iii) independent DNA preparations also starting from different tissues.

### 2.7. 5-methylcytosine immunoprecipitation

Four µg of DNA extracted from both blood and cell lines were incubated with 40 U of *Alu*I restriction endonuclease in a total volume of 20 μl overnight at 37°C and subsequently at 65°C for 20 min to inactivate the endonuclease. The enzymatic DNA digestion allowed us to obtain fragments of appreciable size compared with the random DNA fragmenting by sonication.

5mC immunoprecipitation was carried out using the EpiQuik Methylated DNA Immunoprecipitation (MeDIP) Kit (Epigentek, NY, USA) according to the manufacturer's specifications. First, wells were washed once with Wash Buffer (WB; CP1) and then incubated at RT for 60 min in the presence of 100 µl of Antibody Buffer (AB; CP2) supplemented with 1 µl of 5mC antibody (or 1 µl of Normal Mouse IgG, as a negative control).

After three consecutive washes of the wells with 150 µl CP2, *Alu*I-digested DNA samples, diluted with ChIP Dilution Buffer (CP4), were added into the assay wells. The solution was incubated at RT for 90 min on an orbital shaker to allow DNA binding onto the assay wells. Therefore, the wells were first washed six times with 150 µl of the 1× WB, allowing 2 min on a rocking platform for each wash, followed by the addition of 150 µl 1× tris-EDTA (TE) Buffer. Afterwards, 40 µl of the DNA Release Buffer (DRB) containing proteinase K were added to each well and samples were incubated at 65°C for 15 min. Then, samples were incubated in 40 µl of Reverse Buffer (CP6) at 65°C for 30 min, 150 µl of Binding Buffer (CP7) were subsequently added to the wells, and the released samples, transferred to the F-Spin column, were centrifuged at 14 000 *g* for 20 s. After a centrifugation in the presence of 200 µl of 70% ethanol and two consecutive centrifugations in 90% ethanol, at 14 000 *g* for 30 s, purified DNA was eluted in 15 µl of Elution Buffer (CP8).

CpGenome Universal Unmethylated DNA (Chemicon) and CpGenome Universal Methylated DNA (Chemicon) were used as negative and positive controls, respectively, in order to test the effectiveness of the kit.

### 2.8. 5-hydroxymethylcytosine immunoprecipitation

5hmC immunoprecipitation was carried out using the EpiQuik Hydroxymethylated DNA Immunoprecipitation (hMeDIP) Kit (Epigentek), according to the manufacturer's recommendations. Briefly, 100 µl of antibody buffer (AB) were added to each well of the microplates, followed by adding 1 µl of 5hmC antibody (or 1 µl of Non-Immune IgG, as a negative control) and incubation at RT for 60 min. After removing AB and washing of the wells with 200 µl 1× WB, 1 µg of *Alu*I-digested DNA samples was diluted to 10 ng/µl with hMeDIP Solution (HS) and added into the assay wells to be incubated at RT for 90 min on an orbital shaker. Therefore, the wells were first washed five times with 200 µl of 1× WB, and then, with 200 µl of DNA release buffer (DRB). Afterwards, 40 µl of the DRB containing proteinase K were added to each well and samples were incubated at 60°C for 15 min, followed by an incubation at 95°C for 3 min.

The sensitivity of the methods was estimated by analysing the reference DNA fragment containing 5hmC provided by the kit.

### 2.9. MeDIP/hMeDIP-PCR

Immunoprecipitated methylated and hydroxymethylated DNAs were then used as a template for real-time PCRs carried out using the SYBR Green qPCR Master Mix (Promega) in a StepOne Plus machine (Applied Biosystems). In these reactions, PCR primers specifically amplifying D-loop fragments previously detected by bisulphite sequencing as unmethylated and methylated were used (Supplementary Table S3).

The final PCR mixture (20 μl) contained 1 μl of immunoprecipitated DNA, 1× GoTaq® qPCR Master Mix, 0.2 μM of each primer, and 1× CXR Reference Dye. The thermal profile used for the reaction included a 2-min heat activation of the enzyme at 95°C, followed by 35 cycles of denaturation at 95°C for 15 s and annealing/extension at 58°C for 60 s, followed by melt analysis ramping at 58–95°C. All measurements were taken in the log phase of amplification.

Specific PCR primers were used for verifying the enrichment efficiency of methylated and hydroxymethylated positive control DNAs. Fold enrichment was calculated as the ratio of amplification efficiency of the MeDIP/hMeDIP sample over that of non-immune IgG and then, normalizing the data to the nanograms of immunoprecipitated DNA used in PCR. Six independent triplicate experiments were carried out.

### 2.10. Dot-blot hybridization of MeDIP and hMeDIP samples

Dot-blot hybridization was carried out using the forward and reverse primers reported in Supplementary Table S3 as single-strand oligo probes specifically recognizing the H- and L-strands, respectively.

The probes were labelled to the 3′-OH ends using Terminal Transferase (Roche) according to the manufacturer's protocol. Aliquots of four representative human and mouse MeDIP and hMeDIP samples were immobilized on six different N+ membrane (Amersham Biosciences) strips and fixed with ultraviolet light for 5 min. Then, they were blocked in Detector Block Solution (KPL, Inc.) and incubated at RT for 30 min. After the removal of the solution, each membrane strip was incubated with the mix containing the relevant probe at RT for 30 min in the presence of the Detector Block Solution containing 0.5 μg/ml of horseradish peroxidase (HRP) Streptavidin (KPL, Inc.). After the incubation, the Detector Block Solution was removed and the membrane strips were washed four times with Biotin Wash Solution 1× (KPL, Inc.). Immunoreactivity was determined by means of the ECL chemiluminescence reaction (KPL, Inc.).

### 2.11. Isolation of mitochondrial protein fractions

Mitochondrial extracts were prepared using the Mitochondrial Fractionation Kit (Active Motif). 1.5 × 10^7^ HeLa and 3T3-L1 cells were scraped on ice after the addition of 10 ml of ice-cold 1× phosphate-buffered saline (PBS) and then centrifuged at 600 *g* for 5 min at 4°C. Cell pellets were re-suspended in 5 ml of ice-cold PBS and centrifuged at 600 g for 5 min at 4°C. Then, cell pellets were re-suspended in 250 μl of 1× cytosolic buffer included in the kit and incubated on ice for 15 min. Successively, cell pellets were homogenized and the resulting supernatant was centrifuged at 850 *g* for 20 min at 4°C. After centrifugation, the supernatant, containing the cytosol and the mitochondria, was removed and centrifuged second time at 800 *g* for 10 min at 4°C. Then, the supernatant was again removed and centrifuged at 11 000 *g* for 20 min at 4°C to pellet the mitochondria. Mitochondrial pellets were washed with 100 μl of 1× cytosolic buffer and then centrifuged at 11 000 *g* for 10 min at 4°C. Finally, mitochondrial pellets were lysed by adding 35 μl of complete mitochondria buffer, prepared by adding mitochondria buffer, protease inhibitor cocktail, and dithiothreitol, and by incubating on ice for 15 min.

As a control, whole-protein extracts were obtained according to the standard procedure.

### 2.12. Western blotting of DNMT methyltransferases and TET methylcytosine dioxygenases

Eighty micrograms of whole-protein extracts and 30 µg of cytosolic and mitochondrial protein fractions were resolved on a 7% sodium dodecyl sulphate–polyacrylamide gel electrophoresis and transferred into Hibond-P membranes at 30 V for 2 h at 4°C. Membranes were washed with tris-buffered saline-Tween 20 (TBST) buffer 1× (0.3 mM Tris–HCl, pH 7.5, 2.5 mM NaCl, 0.05% Tween 20) for 10 min and then incubated at RT for 1 h with 5% non-fat dried milk in TBST 1×. Blots were also washed three times with TBST 1× for 10 min and incubated overnight, in TBST containing 1% milk, with anti-DNMT1, DNMT3A, and DNMT3B monoclonal mouse antibodies (1:200), anti-TET1 polyclonal goat antibody (1:200), and anti-TET2 and anti-TET3 polyclonal rabbit antibodies (1:200). Then, anti-mouse, anti-goat, and anti-rabbit (1:5000) antibodies conjugated with HRP (GE Healthcare) were used as secondary antibodies. Immunoreactivity was determined by means of the ECL chemiluminescence reaction (GE Healthcare). Tubulin antibody (1:500) was used as internal control to exclude putative contamination of mitochondrial fraction by cytosolic proteins. Cytochrome c oxidase subunit IV isoform 1 (CoxIV) antibody (1:200) was used as a mitochondrial loading control. All primary antibodies were purchased from Santa Cruz Biotechnology, except for DNMT3A and CoxIV which were from Abcam.

### 2.13. Statistical analysis

Statistical analyses were performed using the SPSS 15.0 statistical software (SPSS, Inc., Chicago, IL, USA). One-way analysis of variance (ANOVA) and Student's *t*-test were adopted, with a significance level defined as *α* = 0.05.

## 3. Results and Discussion

### 3.1. CpG and non-CpG methylation patterns in human mitochondrial D-loop

Bisulphite sequencing was used to investigate the presence of methylated cytosine residues in the Heavy-(H) and Light (L)-strands of the human mitochondrial control region DNA (D-loop).

**Figure 1.**
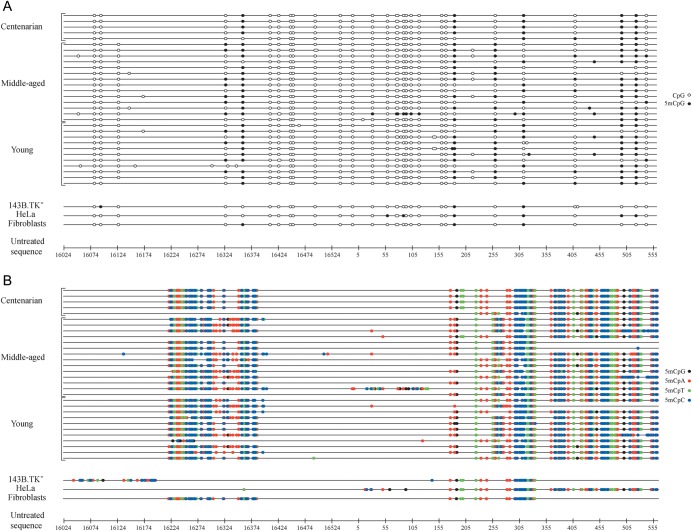
Methylation patterns of the human mitochondrial D-loop in DNA samples from blood and cultured cells. The graphical representation of bisulphite sequencing results was generated by the MethTools software (version 1.2) (http://genome.imb-jena.de/methtools). Bisulphite-generated sequence of each sample (black line) was compared with the untreated sequence of the mitochondrial D-loop, reported here in a base-pair scale. In (A), methylation of cytosine residues located within CpG nucleotides (CpG methylation) is shown. The variability among samples is due to reported polymorphisms (http://www.mitomap.org), which insert or delete cytosines or guanines based on the revised Cambridge reference sequence (rCRS), thus creating/suppressing CpGs. In (B), methylation of cytosine residues located outside of CpG nucleotides (non-CpG methylation) is shown.

Results revealed the presence in the sole L-strand of unconverted cytosines, thus indicating the existence of methylation in the D-loop (Fig. 1A and B and Supplementary File S1). In particular, all analysed clones showed identical methylation patterns, with the majority of the methylated cytosines located outside of CpG nucleotides. The global percentage of methylation (the percentage of methylated cytosines with respect to overall cytosine content of the analysed sequence) was around 35% (values ranging from 32 to 37%, standard deviations of 5%) compared with that at CpG sites equal to 17% (values ranging from 16 to 20%, standard deviations of 2.1%) in DNA samples extracted from human bloods (ANOVA test, *P* < 0.001). Similar patterns were also observed in tumour (HeLa and osteosarcoma 143B.TK^−^) and primary (skin fibroblasts) cell cultures, although they exhibit significant differences in methylation levels at CpG (ANOVA test, *P* = 0.002) and non-CpG sites (ANOVA test, *P* = 0.013). In fact, HeLa cells showed higher overall percentage of methylated cytosines (23 and 27%) compared with fibroblasts (10 and 21%) and osteosarcoma 143B.TK^−^ (10 and 17%) cells, thus suggesting that the observed methylation pattern might be cell-type specific.

All samples, replicate by alternative bisulphite procedures, gave consistent results. As expected, the analysis of both unmethylated control DNA and of the unmethylated purified PCR products of each sub-region revealed the complete absence of unconverted cytosines, confirming that the bisulphite conversion was efficient.

### 3.2. CpG and non-CpG methylation patterns in the mitochondrial D-loop of mouse

**Figure 2.**
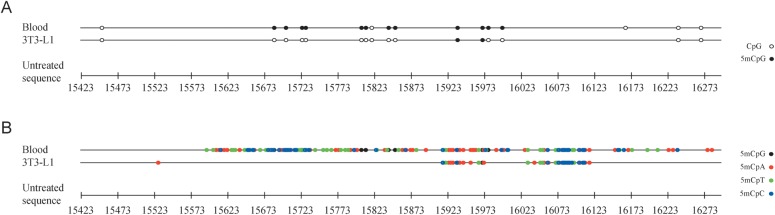
Methylation patterns of the mouse mitochondrial D-loop in DNA samples from blood and cultured cells. In (A), methylation of cytosine residues located within CpG nucleotides (CpG methylation) is shown. In (B), methylation of cytosine residues located outside of CpG nucleotides (non-CpG methylation) is shown.

To outline a broader framework of the mitochondrial D-loop methylation pattern, we extended the analysis to the mouse homologous region. Then, we applied bisulphite sequencing to two DNA genomic samples: the first isolated from whole blood, and the second extracted from 3T3-L1 fibroblast cell cultures. As for humans, the analysis demonstrated not only the presence of methylated residues within the L-strand of the murine mitochondrial D-loop, but also that these residues are preferentially located in non-CpG nucleotides (Fig. 2A and B and Supplementary File S2). However, for both the CpG (ANOVA test, *P* = 0.029) and the non-CpG (ANOVA test, *P* = 0.005) sites, the overall percentage of methylated cytosines appeared to be higher in the blood DNA samples (70.6 and 58.8%, respectively) than in the fibroblasts (12.5 and 17.7%, respectively).

Overall, the overlapping data obtained in both humans and mice seem to indicate that the methylation of the mitochondrial control region could be a general phenomenon and is established across species, at least in the higher eukaryotes, according to similar patterns.

### 3.3. 5-methylcytosine and 5-hydromethylcytosine presence in mtDNA

**Figure 3.**
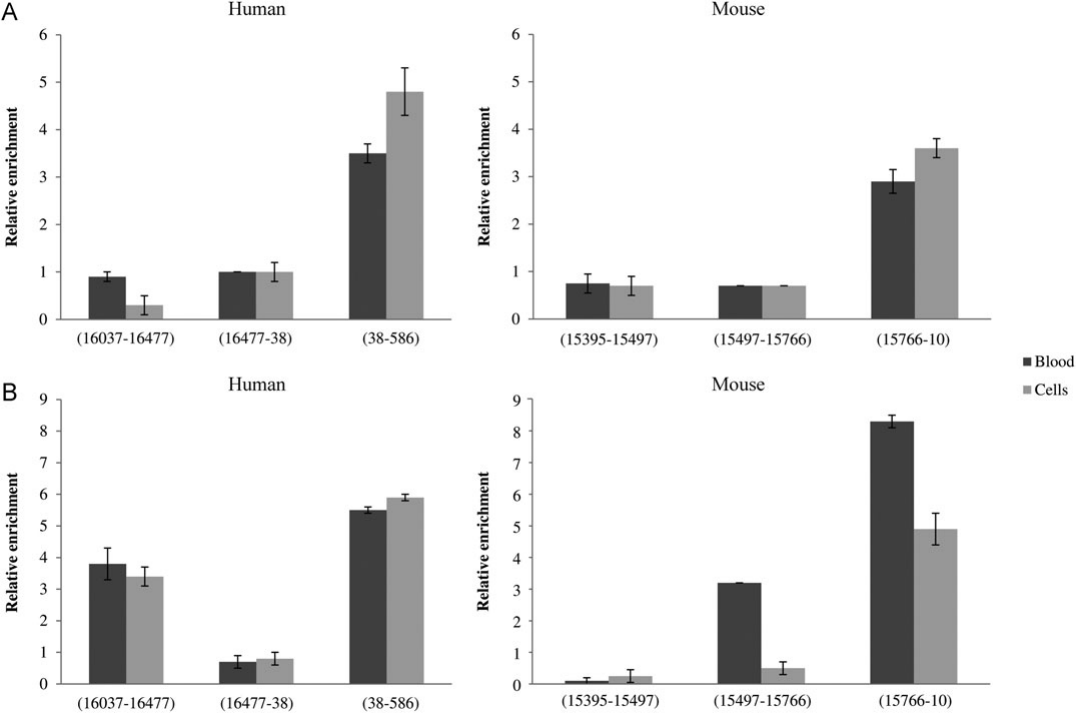
Methylated and hydroxymethylated mtDNA immunoprecipitation. *Alu*I-digested DNA extracted from blood and cultured cells of human and mouse were immunoprecipitated with anti-5mC (A) and anti-5hmC (B). Samples were amplified with primers specific for DNA fragments detected as unmethylated and methylated by bisulphite sequencing. The nucleotide position of these fragments is indicated. Fold enrichment was calculated as the ratio of amplification efficiency of the immunoprecipitated sample over that of non-immune IgG and normalized to the nanograms of immunoprecipitated DNA amount. Data represent the means of six triplicate experiments with standard errors of the mean.

To confirm the methylation status of the mitochondrial D-loop, we determined the presence of 5mC and 5hmC within mtDNA-specific fragments by immunoprecipitation assays, because sodium bisulphite treatment does not distinguish between these two modified bases. In both humans and mice, a significant enrichment for the two bases was observed in those fragments detected by bisulphite sequencing as methylated. In particular, immunoprecipitated DNA fragments were enriched ∼3.5- to 5-folds in humans and 3- to 4-folds in mice for 5mC, relative to the non-immune IgG control and to the immunoprecipitated DNA amount (Fig. 3A). MtDNA immunoprecipitated using anti-5hmC was enriched ∼4- to 6-folds in humans and 3- to 8- folds in mice (Fig. 3B). Noteworthy, the human mtDNA fragment 16 037–16 477 was found to be composed primarily of 5hmC residues. Consistently, with both antibodies, no enrichment was observed for those fragments detected as unmethylated by bisulphite sequencing.

Taken as a whole, the results we obtained demonstrated the presence of both 5mC and 5hmC modifications within the D-loop region with a prevalence of 5hmC.

Finally, DNA probes annealing specifically the H- and L-strands were used in dot-blot hybridization experiments on MeDIP and hMeDIP samples in order to validate the presence of asymmetric methylation. We found positivity for those probes specifically recognizing L-strand, thus demonstrating that this strand is enriched, while H strand did not (Supplementary Fig. S4).

### 3.4. DNA methyltransferases and tet methylcytosine dioxygenases presence within mitochondrial protein fractions

We then questioned whether the enzymes involved in the maintenance and *de novo* methylation of the nuclear DNA could localize within the mitochondria. In addition, we investigated the mitochondrial localization of methylcytosine dioxygenases, reported to be involved in the conversion of 5mC to 5hmC. For this purpose, using human HeLa and murine 3T3-L1 cultured cells as model, we performed immunoblotting assays on mitochondrial protein sub-fractions to detect the presence of DNMT1, DNMT3A, DNMT3B as well as of TET1, TET2, and TET3.

**Figure 4.**
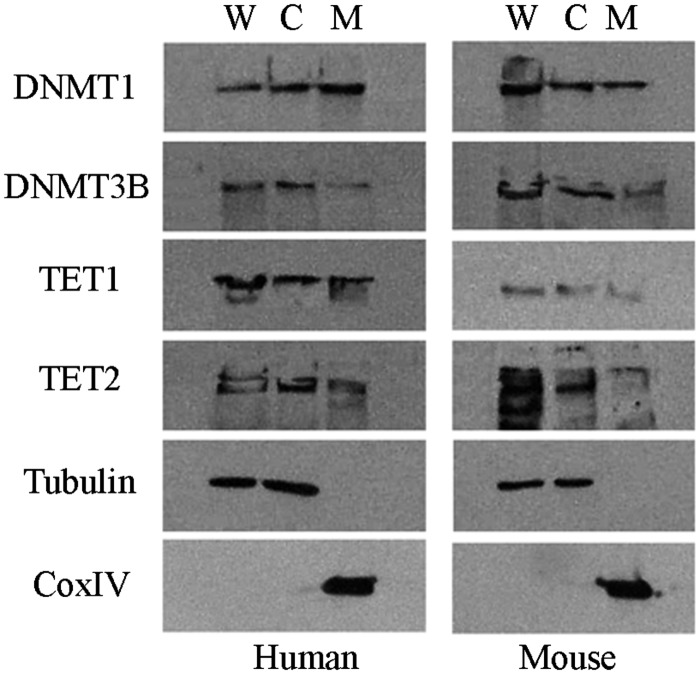
Representative western blot electrophoresis patterns of DNMTs and TETs. Whole cell lysate (W), cytosolic (C), and mitochondrial (M) protein fractions of human HeLa and murine 3T3-L1 cells. CoxIV and tubulin were analysed as mitochondrial- and cytosolic-specific markers, respectively.

As shown in Fig. 4, DNMT1 and very low levels of DNMT3B were observed in the mitochondrial fraction in both humans and mice. As for TET proteins, we found TET1 and TET2 in the mitochondrial fraction of HeLa cells, meanwhile in 3T3-L1 cells only TET1 was detected in the same fraction. On the contrary, we did not find DNMT3A and TET3 in the mitochondrial fraction of the analysed samples (data not shown).

These findings suggest that enzymes involved in nuclear DNA methylation processes can cross the mitochondrial membrane to their final destination inside the mitochondria and, therefore, they might have a potential role in determining the methylation pattern observed in the D-loop.

### 3.5. CpG and non-CpG methylation patterns in the mitochondrial D-loop of *Dnmt1^−/−^*, *Dnmt3a^−/−^*, and *Dnmt3b^−/−^* (TKO) mouse ES cells

To demonstrate whether the methyltransferases located within the mitochondria have a role in determining the methylation patterns observed in the D-loop, we examined the methylation status of the L-strand of this region in mouse ES cells deﬁcient for Dnmt1, Dnmt3a, and Dnmt3b. In these cells, the complete lacking of DNA methylation was checked as reported by Tsumura *et al*.^37^ (Supplementary Fig. S5).

**Figure 5.**
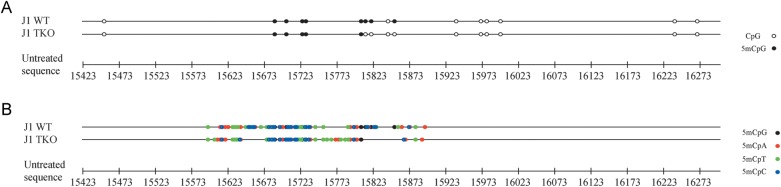
Methylation patterns of the mitochondrial D-loop in DNA samples from wt and *Dnmt1^−/−^*, *Dnmt3a^−/−^*, and *Dnmt3b^−/−^* mouse ES cells. In (A), methylation of cytosine residues located within CpG nucleotides (CpG methylation) is shown. In (B), methylation of cytosine residues located outside of CpG nucleotides (non-CpG methylation) is shown.

Bisulphite sequencing analysis revealed the presence of methylated cytosines within the D-loop region in ∼50% of methylated clones in both the wt and TKO ES DNA samples (Fig. 5A and B and Supplementary File S3). In these clones, the wt DNA sample shows global methylation percentage both at CpG (50%) and non-CpG (28%) sites higher than TKO one (31 and 23%, respectively; ANOVA test, *P* < 0.001). The persistence of methylated cytosines in the D-Loop region of the TKO DNA is consistent with the idea that DNMTs are not the only enzymes involved in the establishment of the reported methylation pattern. What is more, in both samples, the methylation percentage at CpG and non-CpG sites is significantly lower than that of other murine samples previously analysed (ANOVA test, *P* < 0.001). This result, together with the co-presence of both unmethylated and methylated clones, likely indicates that mitochondrial D-loop methylation depends on the differentiation state of cells.

### 3.6. Discussion

The presence of methylated cytosines within the nuclear genome of mammals has been extensively investigated and validated in the last 20 years by several lines of evidences, particularly in relation to their role in the gene expression silencing that explains the inverse correlation between density of methylation within the regulatory sequences of a gene and its transcriptional levels.^16,38–40^

On the contrary, methylation of the mitochondrial genome is largely debated and is far from being elucidated. Indeed, conflicting reports are currently in disagreement over the possible presence of methylated cytosines within mtDNA.^26–35^ Nevertheless, a CpG under-representation in mtDNA, first observed by Pollack *et al*. in mouse and successively by Cardon and McClelland in humans, has always suggested a susceptibility to mutation of this dinucleotide also in the mitochondrial genome and, consequently, to methylation.^31,41,42^

Here, we provide evidence for epigenetic modifications in mtDNA as methylated cytosines were detected within the mitochondrial D-loop in DNA samples extracted from blood and cultured cells of both humans and mice. A peculiar aspect of our data is that the majority of the methylated cytosines were located outside of CpG nucleotides. The presence of non-CpG methylated cytosines might suggest that the limited available data about mtDNA methylation can be attributable to the techniques that have been used to date aimed prevalently at identifying methylated CpG dinucleotides located within recognized sequences of methylation-sensitive restriction enzymes.^27,28^

Of note is also the existence of intercellular variability for both CpG and non-CpG methylation, with higher methylation levels in human blood and HeLa cells with respect to fibroblasts and osteosarcoma cells. The same pattern was observed in mouse, supporting the notion of tissue specificity of DNA methylation. On the other hand, no clear age-related differences were observed.

Although recent findings^35^ have shown the presence of methylation and hydroxymethylation marks (5mC and 5hmC) in mtDNA by immunological and alternative methods, no data have been documented so far about this presence in DNA samples extracted from blood and cultured cells of humans and mice. The results of our study represent the first evidence of the exact localization and the relative abundance of 5hmC, relative to 5mC in the mtDNA from these tissues and cells. Our findings provide also further support to what has already been reported by Shock *et al*., specifically, that epigenetic modifications of cytosines in the mtDNA are likely much more frequent than previously believed.^35,43,44^

It is important to underline the many control experiments we carried out in order to accurately confirm results we obtained. First, we performed different bisulphite treatments, also repeated many months later, to ensure that cytosine conversion was complete. Secondly, we adopted alternative DNA extraction procedures and extended proteinase K treatment for removing any residual protein detrimental to the above conversion. Using both strategies, the observed methylation patterns were similar. Thirdly, we conducted the bisulphite sequencing procedure by using fully unmethylated DNA samples to exclude potential effects on complete bisulphite conversion by specific structural features of the analysed region. On the other hand, we retain that the presence within the analysed region of unconverted cytosines directly adjacent to other converted cytosines represents a good marker of a successful conversion. Finally, both bisulphite sequencing and MeDIP/hMeDIP-PCR assays were carried out also on DNA extracted from osteosarcoma 143B.TK^−^ Rho^0^ cells, completely lacking of mtDNA, in order to exclude spurious amplification of nuclear mitochondrial pseudogenes (NUMTs), with consistent results.^45^

The presence of DNMT1 and much lower levels of DNMT3B enzymes within the mitochondria of the HeLa and 3T3-L1 cells, used as cellular model systems in this study, would suggest that the methylation patterns we observed in the regulatory D-loop are established by these enzymes. In fact, previous studies have demonstrated the involvement of DNMT methyltransferases in the establishment of methylation in both canonical and non-canonical sites.^12,13^ However, the translocation of the DNMTs within the mitochondria is emerging to be tissue-specific, thus we cannot assume that the results observed in HeLa and 3T3-L1 cells are a general phenomenon. However, in spite of these assumptions, *Dnmt1^−/−^*, *Dnmt3a^−/−^*, and *Dnmt3b^−/−^* mouse ES DNA exhibit an analogous methylation pattern, although less marked than that of the wt sample. This finding brought us to believe a non-exclusive involvement of DNMTs in the establishment and maintenance of the methylation patterns we observed within the mitochondrial D-loop. However, more interestingly, the differences in D-loop methylation among the two ES cells with respect to that of other murine cells (blood and fibroblast cells) we analysed as well as the co-presence in ES cells of methylated and unmethylated mtDNA molecules seem to suggest that DNA methylation patterns of adult cells are established at early stage of cell development and can change according to cell and tissue differentiation.

The intra-mitochondrial presence of TET1 and TET2 might imply that the presence of 5hmC within the D-loop could represent an intermediate step in the 5mC demethylation mediated by the above enzymes, thus indirectly regulating the replication or transcription machinery of mtDNA. Therefore, the molecular mechanisms responsible for this methylation, as well as the functional properties of mitochondrial DNMT and TET enzymes within the mitochondria, represent interesting points that deserve further investigation.

Our findings lead us to question on the biological significance of the D-loop methylation. In both human and mouse samples, we identified methylated cytosines in the promoter region of the heavy strand (P_H_) and within conserved sequence blocks (CSBI-III), which are highly conserved sequences located at the 5′-end of the D-loop and considered to be implicated in the processing of the RNA primer during the replication of the H-strand.^46^ Therefore, it is possible to hypothesize that D-loop methylation might play an important role in modulating either replication or transcription of mtDNA, two processes widely described as physically and functionally correlated. Indeed, the regions we found to be methylated are involved in forming the DNA–RNA hybrid due to the transcription of the leading (H)-strand origin. It is of note that the formation of this hybrid occurs to initiate the H-strand replication. Likely, the methylation of L-strand, displaying complementary sequence to RNA primer, may be involved in regulating the formation of the hybrid or stabilizing its persistence. We are currently carrying out experiments that could clarify the functional relevance of epigenetic modifications on the above processes, and the mechanisms which make them possible.

Finally, it is might to speculate that a dense methylation of symmetrical (CpG methylation) and non-symmetrical (non-CpG methylation) sites we observed within the mitochondrial D-loop may resemble typical features RNA-directed DNA methylation (RdDM), so far described only in plants and in fungi.^39,47^ In fact, RdDM occurs specifically along DNA regions that are complementary to RNA, which directs the formation of a putative RNA–DNA duplex. It does not seem a coincidence to have detected the methylation patterns described above within the D-loop, which is a stable triple-helical structure where a RNA sequence forms a hybrid structure with the L-strand.

Taken together, our data provide unequivocal evidence supporting the presence of both methylated and hydroxymethylated cytosines within the human mtDNA control region (D-loop), with an unexpected prevalence in non-CpG moieties. Since both CpG and non-CpG methylated sites were located within the promoter region of the P_H_ and in CSBI-III, it is plausible that epigenetic modifications can regulate replication and/or transcription of the mtDNA. From our data, it also emerges that the peculiar methylation patterns we observed were strictly cell-type dependent, and that the methylation of mitochondrial genome seems to be much more similar to the epigenetic patterns taking place in plants and fungi than those characterizing eukaryotic organisms.

## Supplementary Data

Supplementary data are available at www.dnaresearch.oxfordjournals.org.

## Funding

This work was supported by the European Union's Seventh Framework Programme (FP7/2007–2011, grant number 259679).

## Supplementary Material

Supplementary Data
