# Supplementary material for: The Control Region of Mitochondrial DNA Shows an Unusual CpG and Non-CpG Methylation Pattern
Source: DNA Res. 2013 Jun 26;20(6):537–47. doi: 10.1093/dnares/dst029 (PMC3859322; doi:10.1093/dnares/dst029)
Supplement: Supplementary Data [file supp_20_6_537__index.html]

The Control Region of Mitochondrial DNA Shows an Unusual CpG and Non-CpG Methylation Pattern — Supplementary Data 

# The Control Region of Mitochondrial DNA Shows an Unusual CpG and Non-CpG Methylation Pattern

## 

Supplementary Data

**Files in this Data Supplement:**

- Supplementary Data - Doc file
- Supplementary Figure 1 - doc file
- Supplementary Figure 2 - doc file
- Supplementary Figure 3 - doc file
- Supplementary Figure 4 - tif file
- Supplementary Figure 5 - tif file
- Supplementary Table 1 - doc file
- Supplementary Table 2 - doc file
- Supplementary Table 3 - doc file
