# Supplementary material for: The Control Region of Mitochondrial DNA Shows an Unusual CpG and Non-CpG Methylation Pattern
Source: DNA Res. 2013 Jun 26;20(6):537–47. doi: 10.1093/dnares/dst029 (PMC3859322; doi:10.1093/dnares/dst029)
Supplement: Supplementary Data [file supp_dst029_dst029supp.doc]

**Supplementary Figure S1.** Multiple alignment of complete mitochondrial D-loop sequences of human DNA samples from blood and cultured cells. In red, the revised Cambridge reference sequence (GenBank: NC_012920, [http://www.mitomap.org](http://www.mitomap.org/MITOMAP/HumanMitoSeq)) is reported. Methylated cytosine residues are highlighted in yellow.

**Supplementary Figure S2.** Multiple alignment of complete mitochondrial D-loop sequences of murine DNA samples from blood and cultured cells. In red, the reference sequence (GenBank: NC_005089) is reported. Methylated cytosine residues are highlighted in yellow.

**Supplementary Figure S3.** Multiple alignment of complete mitochondrial D-loop sequences of DNA samples from wild-type (J1 WT) and triple knockout (J1 TKO) mouse ES cells. In red, the reference sequence (GenBank: NC_005089) is reported. Methylated cytosine residues are highlighted in yellow.

**Supplementary Figure S4.** Representative dot-blot assay of human and mouse MeDIP and hMeDIP samples using L- and H- strand specific oligo probes. 1-2 blood samples, 3-4 cultured cell samples. Reverse (Rev) and Forward (For) primers used as labeled probes are indicated. Their sequence is reported in Supplementary Table S3.

**Note. The figure represents 6 different strips (each row corresponds to a strip) joined together before the ECL chemiluminescence reaction and detection.**

**Supplementary Figure S5.** Bisulfite sequencing analysis of LINE 1 repeats in wild-type (J1 WT) and triple knockout (J1 TKO) mouse ES cells. The percentage of methylated cytosines out of total number of CpG sites is indicated. Data represent the mean of three independent experiments with standard error mean.
