# Supplementary material for: The Control Region of Mitochondrial DNA Shows an Unusual CpG and Non-CpG Methylation Pattern
Source: DNA Res. 2013 Jun 26;20(6):537–47. doi: 10.1093/dnares/dst029 (PMC3859322; doi:10.1093/dnares/dst029)
Supplement: Supplementary Data [file supp_dst029_dst029supp_fig1.doc]

1 50

Untreated TTCTTTCATG GGGAAGCAGA TTTGGGTACC ACCCAAGTAT TGACTCACCC

Centenarian TTTTTTTATG GGGAAGTAGA TTTGGGTATT ATTTAAGTAT TGATTTATTT

Centenarian TTTTTTTATG GGGAAGTAGA TTTGGGTATT ATTTAAGTAT TGATTTATTT

Centenarian TTTTTTTATG GGGAAGTAGA TTTGGGTATT ATTTAAGTAT TGATTTATTT

Centenarian TTTTTTTATG GGGAAGTAGA TTTGGGTATT ATTTAAGTAT TGATTTATTT

Centenarian TTTTTTTATG GGGAAGTAGA TTTGGGTATT ATTTAAGTAT TGATTTATTT

Middle_age TTTTTTTATG GGGAAGTAGA TTTGGGTATT ATTTAAGTAT TGATTTATTT

Middle_age TTTTTTTATG GGGAAGTAGA TTTGGGTATT ATTTAAGTAT TGATTTATTT

Middle_age TTTTTTTATG GGGAAGTAGA TTTGGGTATT ATTTAAGTAT TGATTTATTT

Middle_age TTTTTTTATG GGGAAGTAGA TTTGGGTATT ATTTAAGTAT TGATTTATTT

Middle_age TTTTTTTATG GGGAAGTAGA TTTGGGTATT ATTTAAGTAT TGATTTATTT

Middle_age TTTTTTTATG GGGAAGTAGA TTTGGGTATT ATTTAAGTAT TGATTTATTT

Middle_age TTTTTTTATG GGGAAGTAGA TTTGGGTATT GTTTAAGTAT TGATTTATTT

Middle_age TTTTTTTATG GGGAAGTAGA TTTGGGTATT ATTTAAGTAT TGATTTATTT

Middle_age TTTTTTTATG GGGAAGTAGA TTTGGGTATT ATTTAAGTAT TGATTTATTT

Middle_age TTTTTTTATG GGGAAGTAGA TTTGGGTATT ATTTAAGTAT TGATTTATTT

Middle_age TTTTTTTATG GGGAAGTAGA TTTGGGTATT GTTTAAGTAT TGATTTATTT

Middle_age TTTTTTTATG GGGAAGTAGA TTTGGGTATT ATTTAAGTAT TGATTTATTT

Middle_age TTTTTTTATG GGGAAGTAGA TTTGGGTATT ATTTAAGTAT TGATTTATTT

Middle_age TTTTTTTATG GGGAAGTAGA TTTGGGTATT ATTTAAGTAT TGATTTATTT

Young TTTTTTTATG GGGAAGTAGA TTTGGGTATT ATTTAAGTAT TGATTTATTT

Young TTTTTTTATG GGGAAGTAGA TTTGGGTATT ATTTAAGTAT TGATTTATTT

Young TTTTTTTATG GGGAAGTAGA TTTGGGTATT ATTTAAGTAT TGATTTATTT

Young TTTTTTTATG GGGAAGTAGA TTTGGGTATT ATTTAAGTAT TGATTTATTT

Young TTTTTTTATG GGGAAGTAGA TTTGGGTATT ATTTAAGTAT TGATTTATTT

Young TTTTTTTATG GGGAAGTAGA TTTGGGTATT ATTTAAGTAT TGATTTATTT

Young TTTTTTTATG GGGAAGTAGA TTTGGGTATT ATTTAAGTAT TGATTTATTT

Young TTTTTTTATG GGGAAGTAGA TTTGGGTATT ATTTAAGTAT TGATTTATTT

Young TTTTTTTATG GGGAAGTAGA TTTGGGTATT ATTTAAGTAT TGATTTATTT

Young TTTTTTTATG GGGAAGTAGA TTTGGGTATT ATTTAAGTAT TGATTTATTT

Young TTTTTTTATG GGGAAGTAGA TTTGGGTATT ATTTAAGTAT TGATTTATTT

143B.TK- TTTTTTTATG GGGAAGCAGA TTTGGGTACC ACCCAAGTAT TGACTCACCC

HELA TTTTTTTATG GGGAAGTAGA TTTGGGTATT ATTTAAGTAT TGATTTATTT

Fibroblasts TTTTTTTATG GGGAAGTAGA TTTGGGTATT ATTTAAGTAT TGATTTATTT

51 100

Untreated ATCAACAACC GCTATGTATT TCGTACATTA CTGCCAGCCA CCATGAATAT

Centenarian ATTAATAATT GTTATGTATT TTGTATATTA TTGTTAGTTA TTATGAATAT

Centenarian ATTAATAATT GTTATGTATT TTGTATATTA TTGTTAGTTA TTATGAATAT

Centenarian ATTAATAATT GTTATGTATT TTGTATATTA TTGTTAGTTA TTATGAATAT

Centenarian ATTAATAATT GTTATGTATT TTGTATATTA TTGTTAGTTA TTATGAATAT

Centenarian ATTAATAATT GTTATGTATT TTGTATATTA TTGTTAGTTA TTATGAATAT

Middle_age ATTAATAATT GTTATGTATT TTGTATATTA TTGTTAGTTA TTATGAATAT

Middle_age ATTAATAATT GTTATGTATT TTGTATATTA TTGTTAGTTA TTATGAATAT

Middle_age ATTAATAATT GTTATGTATT TTGTATATTA TTGTTAGTTA TTATGAATAT

Middle_age ATTAATAATT GTTATGTATT TTGTATATTA TTGTTAGTTA TTATGAATAT

Middle_age ATTAATAATT GTTATGTATT TTGTATATTA TTGTTAGTTA TTATGAATAT

Middle_age ATTAATAATT GTTATGTATT TTGTATATTA TTGTTAGTTA TTATGAATAT

Middle_age ATTAATAATT GTTATGTATT TTGTATATTA TTGTTAGTTA TTATGAATAT

Middle_age ATTAATAATT GTTATGTATT TTGTATATTA TTGTTAGTTA TTATGAATAT

Middle_age ATTAATAATT GTTATGTATT TTGTATATTA TTGTTAGTTA TTATGAATAT

Middle_age ATTAATAATT GTTATGTATT TTGTATATTA TTGTTAGTTA TTATGAATAT

Middle_age ATTAATAATT GTTATGTATT TTGTATATTA TTGTTAGTTA TTATGAATAT

Middle_age ATTAATAATT GTTATGTATT TTGTATATTA TTGTTAGTTA TTATGAATAT

Middle_age ATTAATAATT GTTATGTATT TTGTATATTA TTGTTAGTTA TTATGAATAT

Middle_age ATTAATAATT GTTATGTATT TTGTATATTA TTGTTAGTTA TTATGAATAT

Young ATTAATAATT GTTATGTATT TTGTATATTA TTGTTAGTTA TTATGAATAT

Young ATTAATAATT GTTATGTATT TTGTATATTA TTGTTAGTTA TTATGAATAT

Young ATTAATAATT GTTATGTATT TTGTATATTA TTGTTAGTTA TTATGAATAT

Young ATTAATAATT GTTATGTATT TTGTATATTA TTGTTAGTTA TTATGAATAT

Young ATTAATAATT GTTATGTATT TTGTATATTA TTGTTAGTTA TTATGAATAT

Young ATTAATAATT GTTATGTATT TTGTATATTA TTGTTAGTTA TTATGAATAT

Young ATTAATAATT GTTATGTATT TTGTATATTA TTGTTAGTTA TTATGAATAT

Young ATTAATAATT GTTATGTATT TTGTATATTA TTGTTAGTTA TTATGAATAT

Young ATTAATAATT GTTATGTATT TTGTATATTA TTGTTAGTTA TTATGAATAT

Young ATTAATAATT GTTATGTATT TTGTATATTA TTGTTAGTTA TTATGAATAT

Young ATTAATAATT GTTATGTATT TTGTATATTA TTGTTAGTTA TTATGAATAT

143B.TK- ATCAATAATT GCTATGTATT TCGTATATTA TTGTTAGTTA TTATGAATAT

HELA ATTAATAATT GTTATGTATT TTGTATATTA TTGTTAGTTA TTATGAATAT

Fibroblasts ATTAATAATT GTTATGTATT TTGTATATTA TTGTTAGTTA TTATGAATAT

101 150

Untreated TGTACGGTAC CATAAATACT TGACCACCTG TAGTACATAA AAACCCAATC

Centenarian TGTATAGTAT TATAAATATT TAATTATTTG TAGTATATAA AAATTTAATT

Centenarian TGTATAGTAT TATAAATATT TAATTATTTG TAGTATATAA AAATTTAATT

Centenarian TGTATAGTAT TATAAATATT TAATTATTTG TAGTATATAA AAATTTAATT

Centenarian TGTATAGTAT TATAAATATT TAATTATTTG TAGTATATAA AAATTTAATT

Centenarian TGTATAGTAT TATAAATATT TAATTATTTG TAGTATATAA AAATTTAATT

Middle_age TGTATGGTAC TATAAATATT TGATTATTTG TAGTATATAA AAATTTAATT

Middle_age TGTATGGTAT TATAAATATT TGATTATTTG TAGTATATAA AAATTTAATT

Middle_age TGTATGGTAT TATAAATATT TGATTATTTG TAGTATATAA AAATTTAATT

Middle_age TGTATGGTAT TATAAATATT TGATTATTTG TAGTATATAA AAATTTAATT

Middle_age TGTATGGTAT TATAAATATT TGATTATTTG TAGTATATAA AAATTTAATT

Middle_age TGTATGGTAT TATAAATATT TGATTGTTTG TAGTATATAA AAATTTAATT

Middle_age TGTATGGTAT TATAAATATT TGATTATTTG TAGTATATAA AAATTTAATT

Middle_age TGTATGGTAT TATAAATATT TAATTATTTG TAGTATATAA AAATTTAATT

Middle_age TGTATGGTAT TATAAATATT TGATTATTTG TAGTATATAA AAATTTAATT

Middle_age TGTATGGTAT TATAAATATT TGATTATTTG TAGTATATAA AAATTTAATT

Middle_age TGTATGGTAT TATAAATATT TGATTATTTG TAGTATATAA AAATTTAATT

Middle_age TGTATGGTAT TATAAATATT TGATTATTTG TAGTATATAA AAATTTAATT

Middle_age TGTATGGTAT TATAAATATT TGATTATTTG TAGTATATAA AAATTTAATT

Middle_age TGTATGGTAT TATAAATATT TGATTGTTTG TAGTATATAA AAATTTAATT

Young TGTATGGTAT TATAAATATT TGATTATTTG TAGTATATAA AAATTTAATT

Young TGTATGGTAT TATAAATATT TGATTATTTG TAGTATATAA AAATTTAATT

Young TGTATGGTGT TATAAATATT TGATTATTTG TAGTATATAA AAATTTATAT

Young TGTATGGTAT TATAAATATT TGATTATTTG TAGTATATAA AAATTTAATT

Young TGTATGGTAT TATAAATATT TGATTATTTG TAGTATATAA AAATTTAATT

Young TGTATGGTAT TATAAATATT TGATTATTTG TAGTATATAA AAATTTAATT

Young TGTATGGTAT TATAAATATT TGATTATTTG TAGTATATAA AAATTTAATT

Young TGTATGGTAT TATAAATATT TGATTATTTG TAGTATATAA AAATTTAATT

Young TGTATAGTAT TATAAATATT TAATTATTTG TAGTATATAA AAATTTAATT

Young TGTATGGTAT TATAAATATT TGATTATTTG TAGTATATAA AAATTTAATT

Young TGTATGGTAT TATAAATATT TGATTATTTG TAGTATATAA AAATTTAATT

143B.TK- TGTATGGTAT CATAAATACT TGACTATTTG TAGTACATAA AAACCCAATC

HELA TGTATGGTAT TATAAATATT TGATTATTTG TAGTATATAA AAATTTAATT

Fibroblasts TGTATGGTAT TATAAATATT TGATTATTTG TAGTATATAA AAATTTAATT

151 200

Untreated CACATCAAAA CCCCCTCCCC ATGCTTACAA GCAAGTACAG CAATCAACCC

Centenarian TAGATTAAAA TTTTTTTTTT ATGTTTATAA GTAAGTATAG TAATCAACCT

Centenarian TAGATTAAAA TTTTTTTTTT ATGTTTATAA GTAAGTATAG TAATCAACCT

Centenarian TAGATTAAAA TTTTTTTTTT ATGTTTATAA GTAAGTATAG TAATCAACCT

Centenarian TAGATTAAAA TTTTTTTTTT ATGTTTATAA GTAAGTATAG TAATCAACCT

Centenarian TAGATTAAAA TTTTTTTTTT ATGTTTATAA GTAAGTATAG TAATTAATTT

Middle_age TATATTAAAA TTTTTTTTTT ATGTTTATAA GTAAGTATAG TAATCAACCC

Middle_age TATATTAAAA TTTTTTTTTT ATGTTTATAA GTAAGTATAG TAATCAACCC

Middle_age TATATTAAAA TTTTTTTTTT ATGTTTATAA GTAAGTATAG TAATCAACCT

Middle_age TGTATTAAAA TTTTTTTTTT ATGTTTATAA GTAAGTATAG TAATTAATTT

Middle_age TATATTAAAA TTTTTTTTTT ATGTTTATAA GTAAGTATAG TAATCAATTC

Middle_age TATATTAAAA TTTTTTTTTT ATGTTTATAA GTAAGTATAG TAATCAACCT

Middle_age TATATTAAAA TTTTTTTTTT ATGTTTATAA GTAAGTATAG TAATCAACCC

Middle_age TATATTAAAA TTTTTTTTTT ATGTTTATAA GTAAGTATAG TAATTAATTT

Middle_age TATATTAAAA TTTTTTTTTT ATGTTTATAA GTAAGTATAG TAATTAACTC

Middle_age TATATTAAAA TTTTTTTTTT ATGTTTATAA GTAAGTATAG TAATTAATCC

Middle_age TATATTAAAA TTTTTTTTTT ATGTTTATAA GTAAGTATAG TAATCAACCC

Middle_age TATATTAAAA TTTTTTTTTT ATGTTTATAA GTAAGTATAG TAATTAATTT

Middle_age TATATTAAAA TTTTTTTTTT ATGTTTATAA GTAAGTATAG TAATCAACCT

Middle_age TATATTAAAA TTTTTTTTTT ATGTTTATAA GTAAGTATAG TAATCAACCT

Young TATATTAAAA TTTTTTTTTT ATGTTTATAA GTAAGTATAG TAATCAACCC

Young TATATTAAAA TTTTTTTTTT ATGTTTATAA GTAAGTATAG TAATTAACCC

Young TAAATTAAAA TTTTTTTTTT ATGTTTATAA GTAAGTATAG TAATCAACCT

Young TATATTAAAA TTTTTTTTTT ATGTTTATAA GTAAGTATAG TAATCAACCT

Young TATATTAAAA TTTTTATTTT ATGTTTATAA GTAAGTATAG TAATCAACCT

Young TATATTAAAA TTTTTATTTT ATGTTTATAA GTAAGTATAG TAATCAACCT

Young TATATTAAAA TTTTTTTTTT ATGTTTATAA GTAAGTATAG TAATCAATTC

Young TATATTAAAA TTTTTTTTTT ATGTTTATAA GTAAGTATAG TAATCAACCC

Young TAGATTAAAA TTTTTTTTTT ATGTTTATAA GTAAGTATAG TAATCAACCT

Young TGTATTAAAA TTTTTTTTTT ATGTTTATAA GTAAGTATAG TAATCAACCC

Young TATATTAAAA TTTTTTTTTT ATGTTTATAA GTAAGTATAG TAATCAACCC

143B.TK- CACATCAAAA CCCCCACCCC ATGTTTATAA GTAAGTATAG TAATTAATTT

HELA TATATTAAAA TTTTTTTTTT ATGTTTATAA GTAAGTATAG TAATTAATTT

Fibroblasts TATATTAAAA TTTTTTTTTT ATGTTTATAA GTAAGTATAG TAATCAACCT

201 250

Untreated TCAACTATCA CACATCAACT GCAACTCCAA AGCCACCCCT CACCCACTAG

Centenarian CCAACTATCA CACATCAACT GCAACTCCAA AGCCACCCCT CACCCACTAG

Centenarian CCAACTATCA CACATCAACT GCAACTCCAA AGCCACCCCT CACCCACTAG

Centenarian CCAACTATCA CACATCAACT GCAACTCCAA AGCCACCCCT CACCCACTAG

Centenarian CCAACTATCA CACATCAACT GCAACTCCAA AGCCACCCCT CACCCACTAG

Centenarian TTAATTATTA TATATTAATT GTAATTTTAA AGTTATTTTT TATTTATTAG

Middle_age CCAACTATCA CACATCAACT GCAACTCCAA AGCCACCTCT CACCTACTAG

Middle_age CCAACTATCA TACATCAACT GCAACTCCAA AGCTATTCTT TATTTATTAG

Middle_age CCAACTATCA CACATCAACT GCAACTCCAA AGCCACCCCT CACCCACTAG

Middle_age CCAACTATCA CACATTAACT GCAACTCCAA AGCCACCCTT CACCCACTAG

Middle_age CCAACTATCA CACATCAACT GCAACTCCAA AGCCACCCCT CACCCACTAG

Middle_age CCAACTATCA CACATCAACT GCAACTCCAA AGCCACCCCT CACCCACTAG

Middle_age TCAACTATCA CACATCAACT GCAACTCCAA AGCCACCCCT CACCCACTAG

Middle_age TTAATTATTA TATATTAATT GTAATTTTAA AGTTATTTTT TATTTATTAG

Middle_age CCAACTATCA CACATCAACT GCAACTCCAA AGCCACCCCT CACTTATTAG

Middle_age CCAACTATCA CACATCAACT GCAACTCCAA AGCCACCCTT CACCTACTAG

Middle_age TCAACTATCA CACATCAACT GCAATTCCAA AGCTATCCCT CATCCACTAG

Middle_age TTAATTATTA TATATTAATT GTAATTTTAA AGTTATTTTT TATTTATTAG

Middle_age CCAACTATCA CACATCAACT GCAACTCCAA AGCCACCCCT CACCCACTAG

Middle_age CCAACTATCA CACATCAACT GCAACTCCAA AGCCACCCCT CACCCACTAG

Young TCAACTATCA CACATCAACT GCAACTCCAA AGCCACCCAT CACCCACTAG

Young CCAACTATCA CACATCAACT GCAACTTCAA AGTTATTCTT CACCCACTAG

Young CCAACTATCA CACATCAACT GCAACTCCAA AGCCACCCCT CACCCACTAG

Young CCAACTATCA CACATCAACT GCAACTCCAA AGCCACCCCT CACCCACTAG

Young CCAACTATCA CACATCAACT GCAACTCCAA AGCCACCCCT CACCCACTAG

Young CCAACTATCA CACATCAACT GCAACTCCAA AGCCACCCCT CACCCACTAG

Young CCAACTATCA CACATCAACT GCAACTCCAA AGCCACCCCT CACCCACTAG

Young CTAACTATCA CACATCAACT GCAACTCCAA AGCCACCCCT CATTCATTAG

Young CCAACTATCA CACATCAACT GCAACTCCAA AGCCACCCCT CACCCACTAG

Young CCAACTATCA CACATCAACT GCAACTCCAA AGCCACCCCT CATCCACTAG

Young TCAACTATCA CACATCAACT GCAACTCCAA AGCCACCCAT CACCCACTAG

143B.TK- TTAATTATTA TATATTAATT GTAATTTTAA AGTTATTTTT TATTTATTAG

HELA TTAATTATTA TATATTAATT GTAATTTTAA AGTTATTTTT TATTTATTAG

Fibroblasts CCAACTATCA CACATCAACT GCAACTCCAA AGCCACCCCT CACCCACTAG

251 300

Untreated GATACCAACA AACCTACCCA CCCTTAACAG TACATAGTAC ATAAAGCCAT

Centenarian GATACCAATA AATTTATCCA CCCTTAACAG TATATAGCAT ATAAAACCAT

Centenarian GATACCAATA AATTTATCCA CCCTTAACAG TATATAGCAT ATAAAACCAT

Centenarian GATACCAATA AATTTATCCA CCCTTAACAG TATATAGCAT ATAAAACCAT

Centenarian GATACCAATA AATTTATCCA CCCTTAACAG TATATAGCAT ATAAAACCAT

Centenarian GATATTAATA AATTTATTTA TTTTTAATAG TATATAGTAT ATAAAGTTAT

Middle_age GATACTAATA AACCTACCCA CCTTTAACAG TACATAGCAC ATAAAACCAT

Middle_age GATATTAATA AATTTACCCA CCCTTAACAG TACATAGCAT ATAAAACTAT

Middle_age GATACCAATA AATTTATCCA CCCTTAACAG TATATAGCAT ATAAAACCAT

Middle_age GATACCAACA AACCTACCCA TCCTTAACAG TACATAGCAC ATAAAACCAT

Middle_age GATACCAACA AACCTATCCA TCCTTAACAG TACATAGCAT ATAAAATCAT

Middle_age GATACCAATA AATTTATCCA CCCTTAACAG TATATAGCAT ATAAAACCAT

Middle_age GATACCAACA AACCTATCCA CCTTTAACAG TACATAGCAT ATAAAATCAT

Middle_age GATATTAATA AATTTATTTA TTTTTAATAG TATATAGTAT ATAAAGTTAT

Middle_age GATACCAACA AACCTACCCA CCCTTAATAG TATATAGTAC ATAAAACCAT

Middle_age GATACCAACA AACCTACCCA CCCTTAACAG TACATAGTAC ATAAAACCAT

Middle_age GATACCAACA AACTTACTCA TCCTTAACAG TACATAGTAC ATAAAGCCAT

Middle_age GATATTAATA AATTTATTTA TTTTTAATAG TATATAGTAT ATAAAGTTAT

Middle_age GATACCAATA AATTTATCCA CCCTTAACAG TATATAGCAT ATAAAACCAT

Middle_age GATACCAATA AATTTATCCA CCCTTAACAG TATATAGCAT ATAAAACCAT

Young GATACCAACA AACCTATCTA TCCTTAATAG TACATAGTAC ATAAAGCCAT

Young GATACCAACA AACCTACCCA CCCTTAATAG TATATAGCAT ATAAAACCAT

Young GATACCAATA AATTTATCCA CCCTTAACAG TATATAGCAT ATAAAACCAT

Young GATACCAATA AATTTATCCA CCCTTAACAG TATATAGCAT ATAAAACCAT

Young GATACCAACA AACCTATCCA TCCTTAACAG TACATAGCAT ATAAAATCAT

Young GATACCAACA AACCTATCCA TCCTTAACAG TACATAGCAT ATAAAATCAT

Young GATACCAACA AACCTATCCA TCCTTAACAG TACATAGCAT ATAAAATCAT

Young GATACCAACA AACCTACCCA CCCTTAATAG TACATAGCAT ATAAAATCAT

Young GATACCAATA AATTTATCCA CCCTTAACAG TATATAGCAT ATAAAACCAT

Young GATACCAACA AACCTACCCA CCCTTAACAG TACATAGTAT ATAAAACCAT

Young GATACCAACA AACCTATCTA TCCTTAATAG TACATAGTAC ATAAAGCCAT

143B.TK- GATATTAATA AATTTATTTA TTTTTAATAG TATATAGTAT ATAAAGTTAT

HELA GATATTAATA AATTTATTTA TTTTTAATAG TATATAGTAT ATAAAGTTAT

Fibroblasts GATACCAATA AATTTATCCA CCCTTAACAG TATATAGCAT ATAAAACCAT

301 350

Untreated TTACCGTACA TAGCACATTA CAGTCAAATC CCTTCTCGTC CCCATGGATG

Centenarian TTATTGTATA TAGTATATTA TAGTCAAATC CCTTCTCGTC CCCATGGATG

Centenarian TTATTGTATA TAGTATATTA TAGTCAAATC CCTTCTCGTC CCCATGGATG

Centenarian TTATTGTATA TAGTATATTA TAGTCAAATC CCTTCTCGTC CCCATGGATG

Centenarian TTATTGTATA TAGTATATTA TAGTCAAATC CCTTCTCGTC CCCATGGATG

Centenarian TTATTGTATA TAGTATATTA TAGTTAAATT TTTTTTTGTT TTTATGGATG

Middle_age TTACCGTATA TAGCATATTA CAGTTAAATC CCTTCTCGTC CCCATGGATG

Middle_age TTACCGTATA TAGCATATTA CAGTTAAATC CCTTCTCGTT TTCATGGATG

Middle_age TTATTGTATA TAGTATATTA TAGTCAAATC CCTTCTCGTC CCCATGGATG

Middle_age TTACCGTACA TAGTACATTA CAGTCAAATC CCTTCTCGTC CCCATGGATG

Middle_age TTATCGTACA TAGCATATTA CAGTCAAATT CTTTCTCGTC CCCATGGATG

Middle_age TTATTGTATA TAGTATATTA TAGTCAAATC CCTTCTCGTC CCCATGGATG

Middle_age TTATCGTACA TAGCATATTA CAGTCAAATT CTTTCTCGTC CCCATGGATG

Middle_age TTATTGTATA TAGTATATTA TAGTTAAATT TTTTTTTGTT TTTATGGATG

Middle_age TTATCGTACA TAGCACATTA CAGTCAAATC CCTTCTCGTC CCCATGGATG

Middle_age TTATCGTACA TAGCACATTA CAGTCAAATC CCTTCTCGTC CCCATGGATG

Middle_age TTATTGTATA TAGCACATTA CAGTCAAATC CCTTCTCGTC CCCATGGATG

Middle_age TTATTGTATA TAGTATATTA TAGTTAAATT TTTTTTTGTT TTTATGGATG

Middle_age TTATTGTATA TAGTATATTA TAGTCAAATC CCTTCTCGTC CCCATGGATG

Middle_age TTATTGTATA TAGTATATTA TAGTCAAATC CCTTCTCGTC CCCATGGATG

Young TTACTGTATA TAGTATATTA TAGTCAAATC CCTTCTCGTC CCCATGGATG

Young TTACCGTACA TAGCATATTA CAGTCAAATC CTTTCTCGTC CCCATGGATG

Young TTATTGTATA TAGTATATTA TAGTCAAATC CCTTCTCGTC CCCATGGATG

Young TTATTGTATA TAGTATATTA TAGTCAAATC CCTTCTCGCC CCCATGGATG

Young TTATCGTACA TAGCATATTA CAGTCAAATT CTTTCTCGTC CCCATGGATG

Young TTATCGTACA TAGCATATTA CAGTCAAATT CTTTCTCGTC CCCATGGATG

Young TTATCGTACA TAGCATATTA CAGTCAAATT CTTTCTCGTC CCCATGGATG

Young TTATTGTATA TAGCATATTA TAGTTAAATC CCTTCTCGTC CCCATGGATG

Young TTATTGTATA TAGTATATTA TAGTCAAATC CCTTCTCGTC CCCATGGATG

Young TTACCGTACA TAGCACATTA CAGTCAAATC TCTTCTCGTC CCCATGGATG

Young TTACTGTATA TAGTATATTA TAGTCAAATC CCTTCTCGTC CCCATGGATG

143B.TK- TTATTGTATA TAGTATATTA TAGTTAAATT TTTTTTTGTT TTTATGGATG

HELA TTATTGTATA TAGTATATTA TAGTTAAATT TTTTCTTGTT TTTATGGATG

Fibroblasts TTATTGTATA TAGTATATTA TAGTCAAATC CCTTCTCGCC CCCATGGATG

351 400

Untreated ACCCCCCTCA GATAGGGGTC CCTTGACCAC CATCCTCCGT GAAATCAATA

Centenarian ACCCCCCTCA GATAGGGGTT TTTTGATTAT TATTTTTTGT GAAATTAATA

Centenarian ACCCCCCTCA GATAGGGGTT TTTTGATTAT TATTTTTTGT GAAATTAATA

Centenarian ACCCCCCTCA GATAGGGGTT TTTTGATTAT TATTTTTTGT GAAATTAATA

Centenarian ACCCCCCTCA GATAGGGGTT TTTTGATTAT TATTTTTTGT GAAATTAATA

Centenarian ATTTTTTTTA GATAGGAGTT TTTTGATTAT TATTTTTTGT GAAATTAATA

Middle_age ACCCTCCTCA GATAGGGGTC CCTTGACTAT TATTTTTTGT GAAATTAATA

Middle_age ACCCCCCTCA GATAGGGGTT TTTTGATTAT TATTTTTTGT GAAATTAATA

Middle_age ACCCCCCTCA GATAGGGGTT TTTTGATTAT TATTTTTTGT GAAATTAATA

Middle_age ACTTTCCTTA GATAGGGGTT TTTTGATTAT TATTTTTTGT GAAATTAATA

Middle_age ATCCCCCTCA GATAGGGGTT TTTTGATTAT TATTTTTTGT GAAATTAATA

Middle_age ACCCCCCTCA GATAGGGGTT TTTTGATTAT TATTTTTTGT GAAATTAATA

Middle_age ACCCTCCTCA GGTAGGGGTC CCTTGACTAT TATTTTTTGT GAAATTAATA

Middle_age ATTTTTTTTA GATAGGGGTT TTTTGATTAT TATTTTTTGT GAAATTAATA

Middle_age ACCTTCCTCA GATAGGGGTC TCTTGATTAT TATTTTTTGT GAAATTAATA

Middle_age ATTTTTTTTA GATAGGGGTT TTTTGATTAT TATTTTTTGT GAAATTAATA

Middle_age ACCCCCCTCA GATAGGGGTT TTTTGATTAT TATTTTTTGT GAAATTAATA

Middle_age ATTTTTTTTA GATAGGGGTT TTTTGATTAT TATTTTTTGT GAAATTAATA

Middle_age ACCCCCCTCA GATAGGGGTT TTTTGATTAT TATTTTTTGT GAAATTAATA

Middle_age ACCCCCCTCA GATAGGGGTT TTTTGATTAT TATTTTTTGT GAAATTAATA

Young ACCCCTTTTA GATAGGGTTC TTTGGATTAT TATTTTTTGT GAAATTAATA

Young ATCCCCCTCA GATAGGGGTT TTTTGATTAT TATTTTTTGT GAAATTAATA

Young ACCCCCCTCA GATAGGGGTT TTTTGATTAT TATTTTTTGT GAAATTAATA

Young ACCCCCCTCA GATAGGGGTT TTTTGATTAT TATTTTTTGT GAAATTAATA

Young ATCCCCCTCA GATAGGGGTT TTTTGATTAT TATTTTTTGT GAAATTAATA

Young ATCCCCCTCA GATAGGGGTT TTTTGATTAT TATTTTTTGT GAAATTAATA

Young ATCCCCCTCA GATAGGGGTT TTTTGATTAT TATTTTTTGT GAAATTAATA

Young ACCCCCCTCA GATAGGGGTC TTTTGATTAT TATTTTTTGT GAAATTAATA

Young ACCCCCCTCA GATAGGGGTT TTTTGATTAT TATTTTTTGT GAAATTAATA

Young ACCCCCCTCA GATAGGGGTC CCTTGATTAT TATTTTTTGT GAAATTAATA

Young ACCCCTTTTA GATAGGGTTC TTTGGATTAT TATTTTTTGT GAAATTAATA

143B.TK- ATTTTTTTTA GATAGGAGTT TTTTGATTAT TATTTTTTGT GAAATTAATA

HELA ATTTTTTTTA GATAGGGGTT TTTTGATTAT TATTTTTTGT GAAATTAATA

Fibroblasts ACCCCCCTCA GATAGGGGTT TTTTGATTAT TATTTTTTGT GAAATTAATA

401 450

Untreated TCCCGCACAA GAGTGCTACT CTCCTCGCTC CGGGCCCATA ACACTTGGGG

Centenarian TTTTGTATAA GAGTGTTATT TTTTTTGTTT TGGGTTTATA ATATTTGGGG

Centenarian TTTTGTATAA GAGTGTTATT TTTTTTGTTT TGGGTTTATA ATATTTGGGG

Centenarian TTTTGTATAA GAGTGTTATT TTTTTTGTTT TGGGTTTATA ATATTTGGGG

Centenarian TTTTGTATAA GAGTGTTATT TTTTTTGTTT TGGGTTTATA ATATTTGGGG

Centenarian TTTTGTATAA GAGTGTTATT TTTTTTGTTT TGGGTTTATA ATATTTGGGG

Middle_age TTTTGTATAA GAGTGTTATT TTTTTTGTTT TGGGTTTATA ATATTTGGGG

Middle_age TTTTGTATAA GAGTGTTATT TTTTTTGTTT TGGGTTTATA ATATTTGGGG

Middle_age TTTTGTATAA GAGTGTTATT TTTTTTGTTT TGGGTTTATA ATATTTGGGG

Middle_age TTTTGTATAA GAGTGTTATT TTTTTTGTTT TGGGTTTATA ATATTTGGGG

Middle_age TTTTGTATAA GAGTGTTATT TTTTTTGTTT TGGGTTTATA ATATTTGGGG

Middle_age TTTTGTATAA GAGTGTTATT TTTTTTGTTT TGGGTTTATA ATATTTGGGG

Middle_age TTTTGTGTAA GAGTGTTATT TTTTTTGTTT TGGGTTTATA ATATTTGGGG

Middle_age TTTTGTATAA GAGTGTTATT TTTTTTGTTT TGGGTTTATA ATATTTGGGG

Middle_age TTTTGTATAA GAGTGTTATT TTTTTTGTTT TGGGTTTATA ATATTTGGGG

Middle_age TTTTGTATAA GAGTGTTATT TTTTTTGTTT TGGGTTTATA ATATTTGGGG

Middle_age TTTTGTATAA GAGTGTTATT TTTTTTGTTT TGGGTTTATA ATATTTGGGG

Middle_age TTTTGTATAA GAGTGTTATT TTTTTTGTTT TGGGTTTATA ATATTTGGGG

Middle_age TTTTGTATAA GAGTGTTATT TTTTTTGTTT TGGGTTTATA ATATTTGGGG

Middle_age TTTTGTATAA GAGTGTTATT TTTTTTGTTT TGGGTTTATA ATATTTGGGG

Young TTTTGTATAA GAGTGTTATT TTTTTTGTTT TGGGTTTATA ATGTTTGGGG

Young TTTTGTATAA GAGTGTTATT TTTTTTGTTT TGGGTTTATA ATATTTGGGG

Young TTTTGTATAA GAGTGTTATT TTTTTTGTTT TGGGTTTATA ATATTTGGGG

Young TTTTGTATAA GAGTGTTATT TTTTTTGTTT TGGGTTTATA ATATTTGGGG

Young TTTTGTGTAA GAGTGTTATT TTTTTTGTTT TGGGTTTATA ATATTTGGGG

Young TTTTGTGTAA GAGTGTTATT TTTTTTGTTT TGGGTTTATA ATATTTGGGG

Young TTTTGTATAA GAGTGTTATT TTTTTTGTTT TGGGTTTATA ATATTTGGGG

Young TTTTGTATAA GAGTGTTATT TTTTTTGTTT TGGGTTTATA ATATTTGGGG

Young TTTTGTATAA GAGTGTTATT TTTTTTGTTT TGGGTTTATA ATATTTGGGG

Young TTTTGTATAA GAGTGTTATT TTTTTTGTTT TGGGTTTATA ATATTTGGGG

Young TTTTGTATAA GAGTGTTATT TTTTTTGTTT TGGGTTTATA ATATTTGGGG

143B.TK- TTTTGTATAA GAGTGTTATT TTTTTTGTTT TGGGTTTATA ATATTTGGGG

HELA TTTTGTATAA GAGTGTTATT TTTTTTGTTT TGGGTTTATA ATATTTGGGG

Fibroblasts TTTTGTATAA GAGTGTTATT TTTTTTGTTT TGGGTTTATA ATATTTGGGG

451 500

Untreated GTAGCTAAAG TGAACTGTAT CCGACATCTG GTTCCTACTT CAGGGTCATA

Centenarian GTAGTTAAAG TGAATTGTAT NTGATATTTG GTTTTTATTT TAGGGTTATA

Centenarian GTAGTTAAAG TGAATTGTAT NTGATATTTG GTTTTTATTT TAGGGTTATA

Centenarian GTAGTTAAAG TGAATTGTAT NTGATATTTG GTTTTTATTT TAGGGTTATA

Centenarian GTAGTTAAAG TGAATTGTAT NTGATATTTG GTTTTTATTT TAGGGTTATA

Centenarian GTAGTTAAAG TGAATTGTAT TTGATATTTG GTTTTTATTT TAGGGTTATA

Middle_age GTAGTTAAAG TGAATTGTAT TTGATATTTG GTTCTTATTT CAGGGTTATA

Middle_age GTAGTTAAAG TGAATTGTAT TTGATATTTG GTTTTTATTT TAGGGTTATA

Middle_age GTAGTTAAAG TGAATTGTAT TTGATATTTG GTTTTTATTT TAGGGTTATA

Middle_age GTAGTTAAAG TGAATTGTAT TTGATATTTG GTTTTTATTT TAGGGTTATA

Middle_age GTAGTTAAAG TGAATTGTAT NTGATATTTG GTTTTTATTT TAGGGTTATA

Middle_age GTAGTTAAAG TGAATTGTAT TTGATATTTG GTTTTTATTT TAGGGTTATA

Middle_age GTAGTTAAAG TGAATTGTAT TTGATATTTG GTTTTTATTT TAGGGTTATA

Middle_age GTAGTTAAAG TGAATTGTAT TTGATATTTG GTTTTTATTT TAGGGTTATA

Middle_age GTAGTTAAAG TGAATTGTAT TTGATATTTG GTTTTTATTT TAGGGTTATA

Middle_age GTAGTTAAAG TGAATTGTAT TTGATGTTTG GTTTTTATTT TAGGGTTATA

Middle_age GTAGTTAGAG TGAATTGTAT TTGATATTTG GTTTTTATTT TAGGGTTATA

Middle_age GTAGTTAAAG TGAATTGTAT TTGATATTTG GTTTTTATTT TAGGGTTATA

Middle_age GTAGTTAAAG TGAATTGTAT TTGATATTTG GTTTTTATTT TAGGGTTATA

Middle_age GTAGTTAAAG TGAATTGTAT TTGATATTTG GTTTTTATTT TAGGGTTATA

Young GTAGTTAAAG TGAATTGTAT TTGATATTTG GTTTTTATTT TAGGGTTATA

Young GTAGTTAAAG TGAATTGTAT TTGATATTTG GTTTTTATTT TAGGGTTATA

Young GTAGTTAAAG TGAATTGTAT TTGATATTTG GTTTTTATTT TAGGGTTATA

Young GTAGTTAAAG TGAATTGTAT TTGATATTTG GTTTTTATTT TAGGGTTATA

Young GTAGTTAAAG TGAATTGTAT TTGATATTTG GTTTTTATTT TAGGGTTATA

Young GTAGTTAAAG TGAATTGTAT TTGATATTTG GTTTTTATTT TAGGGTTATA

Young GTAGTTAAAG TGAATTGTAT TTGATATTTG GTTTTTATTT TAGGGTTATA

Young GTAGTTAAAG TGAATTGTAT TTGATATTTG GTTTTTATTT TAGGGTTATA

Young GTAGTTAAAG TGAACTGTAT TTGATATTTG GTTTTTATTT TAGGGTTATA

Young GTAGTTAAAG TGAATTGTAT TTGATATTTG GTTTTTATTT TAGGGTTATA

Young GTAGTTAAAG TGAATTGTAT TTGATATTTG GTTTTTATTT TAGGGTTATA

143B.TK- GTAGTTAAAG TGAATTGTAT TTGATATTTG GTTTTTATTT TAGGGTTATA

HELA GTAGTTAAAG TGAATTGTAT TTGATATTTG GTTTTTATTT TAGGGTTATA

Fibroblasts GTAGTTAAAG TGAATTGTAT TTGATATTTG GTTTTTATTT TAGGGTTATA

501 550

Untreated AAGCCTAAAT AGCCCACACG TTCCCCTTAA ATAAGACATC ACGATGGATC

Centenarian AAGTTTAAAT AGTTTATATG TTTTTTTTAA ATAAGATATT ATGATGGATT

Centenarian AAGTTTAAAT AGTTTATATG TTTTTTTTAA ATAAGATATT ATGATGGATT

Centenarian AAGTTTAAAT AGTTTATATG TTTTTTTTAA ATAAGATATT ATGATGGATT

Centenarian AAGTTTAAAT AGTTTATATG TTTTTTTTAA ATAAGATATT ATGATGGATT

Centenarian AAGTTTAAAT AGTTTATATG TTTTTTTTAA ATAAGATATT ATGATGGATT

Middle_age AAGTTTAAAT AGTTTATATG TTTTTTTTAA ATAAGATATT ATGATGGATT

Middle_age AAGTTTAAAT AGTTTATATG TTTTTTTTAA ATAAGATATT ATGATGGATT

Middle_age AAGTTTAAAT AGTTTATATG TTTTTTTTAA ATAAGATATT ATGATGGATT

Middle_age AAATTTAAAT AGTTTATATG TTTTTTTTAA ATAAGATATT ATGATGGATT

Middle_age AAGTTTAAAT AGTTTATATG TTTTTTTTAA ATAAGATATT ATGATGGATT

Middle_age AAGTTTAAAT AGTTTATATG TTTTTTTTAA ACAAGATATT ATGATGGATT

Middle_age AAGTTTAAAT AGTTTATATG TTTTTTTTAA ATAAGACATC ATGATGGATT

Middle_age AAGTTTAAAT AGTTTATATG TTTTTTTTAA ATAAGATATT ATGATGGATT

Middle_age AAGTTTAAAT AGTTTATATG TTTTTTTTAA ATAAGATATT ATGATGGATT

Middle_age AAGTTTAAAT AGTTTATATG TTTTTTTTAA ATAAGATATT ATGATGGATT

Middle_age AAGTTTAAAT AGTTTATATG TTTTTTTTAA ATAAGATATT ATGATGGATT

Middle_age AAGTTTAAAT AGTTTATATG TTTTTTTTAA ATAAGATATT ATGATGGATT

Middle_age AAGTTTAAAT AGTTTATATG TTTTTTTTAA ATAAGATATT ATGATGGATT

Middle_age AAGTTTAAAT AGTTTATATG TTTTTTTTAA ATAAGATATT ATGATGGATT

Young AAGTTTAAAT AGTTTATATG TTTTTTTTAA ATAAGATATT ATGATGGATT

Young AAGTTTAAAT AGTTTATATG TTTTTTTTAA ATAAGATATT ATGATGGATT

Young AAGTTTAAAT AGTTTATATG TTTTTTTTAA ATAAGATATT ATGATGGATT

Young AAGTTTAAAT AGTTTATATG TTTTTTTTAA ATAAGATATT ATGATGGATT

Young AAGTTTAAAT AGTTTATATG TTTTTTTTAA ATAAGATATT ATGATGGATT

Young AAGTTTAAAT AGTTTATATG TTTTTTTTAA ATAAGATATT ATGATGGATT

Young AAGTTTAAAT AGTTTATATG TTTTTTTTAA ATAAGATATT ATGATGGATT

Young AAGTTTAAAT AGTTTATATG TTTTTTTTAA ATAAGATATT ATGATGGATT

Young AAGTTTAAAT AGTTTATATG TTTTTTTTAA ATAAGATATT ATGATGGATT

Young AAGTTTAAAT AGTTTATATG TTTTTTTTAA ATAAGATATT ATGATGGATT

Young AAGTTTAAAT AGTTTATATG TTTTTTTTAA ATAAGATATT ATGATGGATT

143B.TK- AAGTTTAAAT AGTTTATATG TTTTTTTTAA GTAAGATATT ATGATGGATT

HELA AAGTTTAAAT AGTTTATATG TTTTTTTTAA ATAAGATATT ATGATGGATT

Fibroblasts AAGTTTAAAT AGTTTATATG TTTTTTTTAA ATAAGATATT ATGATGGATT

551 600

Untreated ACAGGTCTAT CACCCTATTA ACCACTCACG GGAGCTCTCC ATGCATTTGG

Centenarian ATAGGTTTAT TATTTTATTA ATTATTTATG GGAGTTTTTT ATGTATTTGG

Centenarian ATAGGTTTAT TATTTTATTA ATTATTTATG GGAGTTTTTT ATGTATTTGG

Centenarian ATAGGTTTAT TATTTTATTA ATTATTTATG GGAGTTTTTT ATGTATTTGG

Centenarian ATAGGTTTAT TATTTTATTA ATTATTTATG GGAGTTTTTT ATGTATTTGG

Centenarian ATAGGTTTAT TATTTTATTA ATTATTTATG GGAGTTTTTT ATGTATTTGG

Middle_age ATAGGTTTAT TATTTTATTA ATCATTTATG GGAGTTTTTT ATGTATTTGG

Middle_age ATAGGTTTAT TATTTTATTA ATTATTTATG GGAGTTTTTT ATGTATTTGG

Middle_age ATAGGTTTAT TATTTTATTA ATTATTTATG GGAGTTTTTT ATGTATTTGG

Middle_age ATAGGTTTAT TATTTTATTA ATTATTTATG GGAGTTTTTT ATGTATTTGG

Middle_age ATAGGTTTAT TATTTTATTA ATTATTTATG GGAGTTTTTT ATGTATTTGG

Middle_age ATAGGTTTAT TATTTTATTA ATTATTTATG GGAGTTTTTT ATGTATTTGG

Middle_age ATAGGTTTAT TATCCTATTA ACCACTCACG GGAGCTCTCT ATGCATTTGG

Middle_age ATAGGTTTAT TGTTTTATTA ATTATTTATG GGAGTTTTTT ATGTATTTGG

Middle_age ATAGGTTTAT TATTTTATTA ATTATTTATG GGAGTTTTTT ATGTATTTGG

Middle_age ATAGGTTTAT TATTTTATTA ATTATTTATG GGAGTTTTTT ATGTATTTGG

Middle_age ATAGGTTTAT TATTTTATTA ATCATTTATG GGAGTTTTTT ATGTATTTGG

Middle_age ATAGGTTTAT TATTTTATTA ATTATTTATG GGAGTTTTTT ATGCATTTGG

Middle_age ATAGGTTTAT TATTTTATTA ATTATTTATG GGAGTTTTTT ATGTATTTGG

Middle_age ATAGGTTTAT TATTTTATTA ATTATTTATG GGAGTTTTTT ATGTATTTGG

Young ATAGGTTTAT TATTTTATTA ATTATTTATG GGAGTTTTTT ATGTATTTGG

Young ATAGGTTTAT TATTTTATTA ATTATTTATG GGAGTTTTTT ATGTATTTGG

Young ATAGGTTTAT TATTTTATTA ATTATTTATG GGAGTTTTTT ATGTATTTGG

Young ATAGGTTTAT TATTTTATTA ATTATTTATG GGAGTTTTTT ATGTATTTGG

Young ATAGGTTTAT TATTTTATTA ATTATTTATG GGAGTTTTTT ATGTATTTGG

Young ATAGGTTTAT TATTTTATTA ATTATTTATG GGAGTTTTTT ATGTATTTGG

Young ATAGGTTTAT TTTTTTATTA ATTATTTATG GGAGTTTTTT ATGTATTTGG

Young ATAGGTTTAT TATTTTATTA ATTATTTATG GGAGTTTTTT ATGTATTTGG

Young ATAGGTTTAT TATTTTATTA ATTATTTATG GGAGTTTTTT ATGTATTTGG

Young ATAGGTTTAT TATTTTATTA ATCATTTATG GGAGTTTTTT ATGTATTTGG

Young ATAGGTTTAT TATTTTATTA ATTATTTATG GGAGTTTTTT ATGTATTTGG

143B.TK- ATAGGTTTAN TANNNNATTA ATTANNTATG GGAGTTTTTT ATGTATNNGG

HELA ATAGGTTTAT CATCTTATTA ACCATTTATG GGAGTTTTTT ATGCATTTGG

Fibroblasts ATAGGTTTAT TATTTTATTA ATTATTTATG GGAGTTTTTT ATGTATTTGG

601 650

Untreated TATTTTCGTC TGGGGGGTAT GCACGCGATA GCATTGCGAG ACGCTGGAGC

Centenarian TATTTTTGTT TGGGGGGTGT GTATGTGATA GTATTGTGAG ATGTTGGAGT

Centenarian TATTTTTGTT TGGGGGGTGT GTATGTGATA GTATTGTGAG ATGTTGGAGT

Centenarian TATTTTTGTT TGGGGGGTGT GTATGTGATA GTATTGTGAG ATGTTGGAGT

Centenarian TATTTTTGTT TGGGGGGTGT GTATGTGATA GTATTGTGAG ATGTTGGAGT

Centenarian TATTTTTGTT TGGGGGGTGT GTATGTGATA GTATTGTGAG ATGTTGGAGT

Middle_age TATTTTTGTT TGGGGGGTGT GTATGTGATA GTATTGTGAG ATGTTGGAGT

Middle_age TATTTTTGTT TGGGGGGTGT GTATGTGATA GTATTGTGAG ATGTTGGAGT

Middle_age TATTTTTGTT TGGGGGGTAT GTATGTGATA GTATTGTGGG ATGTTGGAGT

Middle_age TATTTTTGTT TGGGGGGTGT GTATGTGATA GTATTGTGAG ATGTTGGAGT

Middle_age TATTTTTGTT TGGGGGGTGT GTATGTGATA GTATTGTGAG ATGTTGGAGT

Middle_age TATTTTTGTT TGGGGGGTGT GTATGTGATA GTATTGTGAG ATGTTGGAGT

Middle_age TATTTTTGTT TGGGGGGTGT GCACGCGATA GCATTGCGAG ACGTTGGAGC

Middle_age TATTTTTGTT TGGGGGGTGT GTATGTGATA GTATTGTGAG ATGTTGGAGT

Middle_age TATTTTTGTT TGGGGGGTGT GTATGTGATA GTATTGTGAG ATGTTGGAGT

Middle_age TATTTTTGTT TGGGGGGTAT GTATGTGATA GTATTGTGAG ATGTTGGAGT

Middle_age TATTTTTGTT TGGGGGGTGT GTATGTGATA GTATTGTGAG ATGTTGGAGT

Middle_age TATTTTTGTT TGGGGGGTGT GTATGTGACA GTATTGTGAG ATGTTGGAGT

Middle_age TATTTTTGTT TGGGGGGTGT GTATGTGATA GTATTGTGAG ATGTTGGAGT

Middle_age TATTTTTGTT TGGGGGGTGT GTATGTGATA GTATTGTGAG ATGTTGGAGT

Young TATTTTTGTT TGGGGGGTAT GTATGTGATA GTATTGTGAG ATGTTGGAGT

Young TATTTTTGTT TGGGGGGTAT GTATGTGATA GTATTGTGAG ATGTTGGAGT

Young TATTTTTGTT TGGGGGGTAT GTATGTGATA GTATTGAGAG ATGTTGGAGT

Young TATTTTTGTT TGGGGGGTGT GTATGTGATA GTATTGTGAG ATGTTGGAGT

Young TATTTTTGTT TGGGGGGTGT GTATGTGATA GTATTGTGAG ATGTTGGAGT

Young TATTTTTGTT TGGGGGGTAT GTATGTGATA GTATTGTGAG ATGTTGGAGT

Young TATTTTTGTT TGGGGGGTGT GTATGTGATA GTATTGTGAG ATGTTGGAGT

Young TGTTTTTGTT TGGGGGGTAT GTATGTGATA GTATTGTGAG ATGTTGGAGT

Young TATTTTTGTT TGGGGGGTGT GTATGTGATA GTATTGTGAG ATGTTGGAGT

Young TATTTTTGTT TGGGGGGTGT GTATGTGATA GTATTGTGAG ATGTTGGAGT

Young TATTTTTGTT TGGGGGGTGT GTATGTGATA GTGTTGTGGG ATGTTGGAGT

143B.TK- NATNNNTGTT TGGGGGGTGT GTATGTGATA GTATTGTGAG ATGTTGGAGT

HELA TATTTTCGTT TGGGGGGTGT GTATGTGATA GTATTGCGAG ATGTTGGAGT

Fibroblasts TATTTTTGTT TGGGGGGTAT GTATGTGATA GTATTGTGAG ATGTTGGAGT

651 700

Untreated CGGAGCACCC TATGTCGCAG TATCTGTCTT TGATTCCTGC CTCATCCTAT

Centenarian TGGAGTATTT TATGTTGTAG TATTTGTTTT TGATTTTTGT TTTATTTTAT

Centenarian TGGAGTATTT TATGTTGTAG TATTTGTTTT TGATTTTTGT TTTATTTTAT

Centenarian TGGAGTATTT TATGTTGTAG TATTTGTTTT TGATTTTTGT TTTATTTTAT

Centenarian TGGAGTATTT TATGTTGTAG TATTTGTTTT TGATTTTTGT TTTATTTTAT

Centenarian TGGAGTATTT TATGTTGTAG TATTTGTTTT TGATTTTTGT TTTATTTTAT

Middle_age TGGAGTATTT TATGTTGTAG TGTTTGTTTT TGATTTTTGT TTTATTTTAT

Middle_age TGGAGTATTT TATGTTGTAG TATTTGTTTT TGATTTTTGT TTTATTTTAT

Middle_age TGGAGTATTT TATGTTGTAG TATTTGTTTT TGATTTTTGT TTTATTTTAT

Middle_age TGGAGTATTT TATGTTGTAG TATTTGTTTT TGATTTTTGT TTTATTTTAT

Middle_age TGGAGTATTT TATGTTGTAG TATTTGTTTT TGATTTTTGT TTTATTTTAT

Middle_age TGGAGTATTT TATGTTGTAG TATTTGTTTT TGATTTTTGT TTTATTTTAT

Middle_age CGGAGCACTC TATGTCGCAG TATCTGTCTT TGATTTTTGT TTTATTTTAT

Middle_age TGGAGTATTT TATGTTGTAG TATTTGTTTT TGATTTTTGT TTTATTTTAT

Middle_age TGGAGTATTT TATGTTGTAG TATTTGTTTT TGATTTTTGT TTTATTTTAT

Middle_age TGGAGTATTT TATGTTGTAG TATTTGTTTT TGATTTTTGT TTTATTTTAT

Middle_age TGGAGTATTT TNNGTTGTAG TGTTTGTTTT TGATTTTTGT TTTATTTTAT

Middle_age TGGAGTATTT TATGTTGTAG TATTTGTTTT TGATTTTTGT TTTATTTTAT

Middle_age TGGAGTATTT TATGTTGTAG TATTTGTTTT TGATTTTTGT TTTATTTTAT

Middle_age TGGAGTATTT TATGTTGTAG TATTTGTTTT TGATTTTTGT TTTATTTTAT

Young TGGAGTATTT TATGTTGTAG TATTTGTTTT TGATTTTTGT TTTATTTTAT

Young TGGAGTATTT TATGTTGTAG TATTTGTTTT TGATTTTTGT TTTATTTTAT

Young TGGAGTATTT TATGTTGTAG TATTTGTTTT TGATTTTTGT TTTGTTGTTT

Young TGGAGTATTT TATGTTGTAG TATTTGTTTT TGATTTTTGT TTTATTTTAT

Young TGGAGTATTT TATGTTGTAG TATTTGTTTT TGATTTTTAT TTTATTTTAT

Young TGGAGTATTT TATGTTGCAG TATTTGTTTT TGATTTTTGT TTTATTTTAT

Young TGGAGTATTT TATGTTGTAG TATTTGTTTT TGATTTTTGT TTTATTTTAT

Young TGGAGTATTT TATGTTGTAG TATTTGTTTT TGATTTTTGT TTTATTTTAT

Young TGGAGTATTT TATGTTGTAG TATTTGTTTT TGATTTTTGT TTTATTTTAT

Young TGGAGTATTT TATGTTGTAG TATTTGTTTT TGATTTTTGT TTTATTTTAT

Young TGGAGTATTT TATGTTGGTA TATTTGTTTT TGATTTTTGT TTTGTTGTTT

143B.TK- TGGAGTATTT TATGTTGTAG TATTTGTTTT TGATTCTTGT TTTATTTTAT

HELA TGGAGTATTT TATGTTGTAG TATTTGTTTT TGATTTTTGT TTTATTTTAT

Fibroblasts TGGAGTATTT TATGTTGTAG TATTTGTTTT TGATTTTTGT TTTATTTTAT

701 750

Untreated TATTTATCGC ACCTACGTTC AATATTACAG GCGAACATAC TTACTAAAGT

Centenarian TATTTATTGT ATTTATGTTC AATATTATAG GCGAATATAC TTACTAAAGT

Centenarian TATTTATTGT ATTTATGTTC AATATTATAG GCGAATATAC TTACTAAAGT

Centenarian TATTTATTGT ATTTATGTTC AATATTATAG GCGAATATAC TTACTAAAGT

Centenarian TATTTATTGT ATTTATGTTC AATATTATAG GCGAATATAC TTACTAAAGT

Centenarian TATTTATTGT ATTTATGTTT AATATTATAG GTGAATATAT TTATTAAAGT

Middle_age TATTTATTGT ATTTATGTTC AATACTACAG ACGGATATAT TTATTAAAGT

Middle_age TATTTATTGT ATTTATGTTT AATATTATAG GTGAATATAT TTATTAAAGT

Middle_age TATTTATTGT ATTTATGTTT AATATTATAG GTGAATATAT TTATTAAAGT

Middle_age TATTTATTGT ATTTATGTTC AATATTACAG ACGGATATAT TTATTAAAGT

Middle_age TATTTATTGT ATTTATGTTT AATATTATAG GTGAATATAT TTATTAAAGT

Middle_age TATTTATTGT ATTTATGTTC AATATTACAG ACGGATATAT TTATTAAAGT

Middle_age TATTTATTGT ATTTATGTTT AATATTATAG GTGAATATAT TTATTAAAGT

Middle_age TATTTATTGT ATTTATGTTC AATATTACAG ACGGATATAT TTATTAAAGT

Middle_age TATTTATTGT ATTTATGTTC AATATTACAG ACGGATATAT TTATTAAAGT

Middle_age TATTTATTGT ATTTATGTTC AATATTACAG ACGGATATAT TTATTAAAGT

Middle_age TATTTATTGT ATTTATGTTC AATATTACAG ACGGATATAT TTATTAAAGT

Middle_age TATTTATTGT ATTTATGTTC AATATTATAG GCGAATATAC TTACTAAAGT

Middle_age TATTTATTGT ATTTATGTTC AATATTACAG ACGGATATAT TTATTAAAGT

Middle_age TATTTATTGT ATTTATGTTC AATATTACAG ACGGATATAT TTATTAAAGT

Young TATTTATTGT ATTTATGTTT AATATTATAG GTGAATATAT TTATTAAAGT

Young TATTTATTGT ATTTATGTTC AATATTACAG ACGGATATAT TTATTAAAGT

Young TATTTATTGT ATTTATGTTC AATATTACGG ACGGATATAT TTATTAAAGT

Young TATTTATTGT ATTTATGTTC AATATTACAG ACGGATATAT TTATTAAAGT

Young TATTTATTGT ATTTATGTTC AATATTACAG ACGGATANAT TTATTAAAGT

Young TATTTATTGT ATTTATGTTT AATATTATAG GTGAATATAT TTATTAAAGT

Young TATTTATTGT ATTTATGTTC AATATTACAG GCGAATATAT TTATTAAAGT

Young TATTTATTGT ATTTATGTTC AATATTACAG ACGGATATAT TTATTAAAGT

Young TATTTATTGT ATTTATGTTT AATATTATAG GTGAGTATAT TTATTAAAGT

Young TATTTATTGT ATTTATGTTT AATATTACAG GTGAGTATAT TTATTAAAGT

Young TATTTATTGT ATTTATGTTT AATATTACAG GCGAATATAT TTATTAAAGT

143B.TK- TATTTATTGT ATTTATGTTC AATATTATAG GCGAATATAC TTACTAAAGT

HELA TATTTATTGT ATTTATGTTC AATATTATAG GCGAATATAC TTACTAAAGT

Fibroblasts TATTTATTGT ATTTATGTTC AATATTATAG GCGAATATAC TTACTAAAGT

751 800

Untreated GTGTTAATTA ATTAATGCTT GTAGGACATA ATAATAACAA TTGAATGTCT

Centenarian GTGTTAATTA ATTAATGCTT TTAGGACATA ATAATAACAA TTGAATGTTT

Centenarian GTGTTAATTA ATTAATGCTT TTAGGACATA ATAATAACAA TTGAATGTTT

Centenarian GTGTTAATTA ATTAATGCTT TTAGGACATA ATAATAACAA TTGAATGTTT

Centenarian GTGTTAATTA ATTAATGCTT TTAGGACATA ATAATAACAA TTGAATGTTT

Centenarian GTGTTAGTTA ATTAATGCTT GTAGGACATA ATAATAACAA TTGAATGTCT

Middle_age GTGTTAATTA ATTAACGCTT GTAGGATATA ATAATAATAA TTGAATGTCT

Middle_age GTGTTAATTA ATTAATGCTT GTAGGACATA ATAATAACAA TTGAATGTCT

Middle_age GTGTTAGTTA ATTAATGCTT GTAGGACATA ATAATAACAA TTGAATGTCT

Middle_age GTGTTAATTA ATTAACGCTT GTAGGATATA ATAATAATAA TTGAATGTCT

Middle_age GTGTTAGTTA ATTAATGCTT GTAGGACATA ATAATAACAA TTGAATGTCT

Middle_age GTGTTAATTA ATTAATGCTT GTAGGATATA ATAATAATAA TTGAATGTCT

Middle_age GTGTTAGTTA ATTAATGCTT GTAGGACATA ATAATAACAA TTGAATGTCT

Middle_age GTGTTAATTA ATTAATGCTT GTAGGATATA ATAATAATAA TTGAATGTCT

Middle_age GTGTTAATTA ATTAATGCTT GTAGGATATA ATAATAATAA TTGAATGTCT

Middle_age GTGTTAATTA ATTAACGCTT GTAGGATATA ATAATAATAA TTGAATGTCT

Middle_age GTGTTAATTA ATTAACGCTT GTAGGATATA ATAATAATAA TTGAATGTCT

Middle_age GTGTTAATTA ATTAATGCTT TTAGGACATA ATAATAACAA TTGAATGTTT

Middle_age GTGTTAATTA ATTAATGCTT GTAGGATATA ATAATAATAA TTGAATGTCT

Middle_age GTGTTAATTA ATTAACGCTT GTAGGATATA ATAATAATAA TTGAATGTCT

Young GTGTTAATTA ATTAATGCTT GTAGGACATA ATAATAACAA TTGAATGTCT

Young GTGTTAATTA ATTAATGCTT GTAGGATATA ATAATAATAA TTGAATGTCT

Young GTGTTAATTA ATTAATGCTT GTAGGATATA ATAATAATAA TTGAATGTCT

Young GTGTTAATTA ATTAACGCTT GTAGGATATA ATAATAATAA TTGAATGTCT

Young GTGTTAATTA ATTAATGCTT GTAGGATATA ATAATAATAA TTGAATGTCT

Young GTGTTAATTA ATTAATGCTT GTAGGACATA ATAATAACAA TTGAATGTCT

Young GTGTTAATTA ATTAACGCTT GTAGGATATA ATAATAATAA TTGAATGTCT

Young GTGTTAATTA ATTAACGCTT GTAGGATATA ATAATAATAA TTGAATGTCT

Young GTGTTAGTTA ATTAATGCTT GTAGGACATA ATAATAACAA TTGAATGTCT

Young GTGTTAATTA ATTAATGCTT GTAGGATATA ATAATAATAA TTGAATGTCT

Young GTGTTAATTA ATTAATGCTT GTAGGACATA ATAATAACAA TTGAATGTTT

143B.TK- GTGTTAATTA ATTAATGCTT TTAGGACATA ATAATAACAA TTGAATGTTT

HELA GTGTTAATTA ATTAATGCTT TTAGGACATA ATAATAACAA TTGAATGTTT

Fibroblasts GTGTTAATTA ATTAATGCTT TTAGGACATA ATAATAACAA TTGAATGTTT

801 850

Untreated GCACAGCCAC TTTCCACACA GACATCATAA CAAAAAATTT CCACCAAACC

Centenarian GTATAGTTGT TTTTTATATA GATATCATAA CAAAAAATTT CCACCAAACC

Centenarian GTATAGTTGT TTTTTATATA GATATCATAA CAAAAAATTT CCACCAAACC

Centenarian GTATAGTTGT TTTTTATATA GATATCATAA CAAAAAATTT CCACCAAACC

Centenarian GTATAGTTGT TTTTTATATA GATATCATAA CAAAAAATTT CCACCAAACC

Centenarian GCACAGTTGC TTTTTATATA GATATCATAA CAAAAAATTT CCACCAAATT

Middle_age GCACAGCCGC TTTCCACACA GATATTATAA CAAAAAATTT CCACCAAACC

Middle_age GCACAGTTGC TTTTTATATA GATATCATAA CAAAAAATTT CCACCAAATT

Middle_age GCACAGTTGC TTTTCATATA GATATCATAA CAAAAAATTT CCACCAAATT

Middle_age GCACAGCCGC TTTCCACACA GATATTATAA CAAAAAATTT CCACCAAACC

Middle_age GCACAGTTGC TTTTTATATA GATATCATAA CAAAAAATTT CCACCAAATT

Middle_age GCACAGCCGC TTTCCACACA GATATTATAA CAAAAAATTT CCACCAAACC

Middle_age GCACAGTTGC TTTTTATATA GATATCATAA CAAAAAATTT CCACCGAATT

Middle_age GCACAGCCGC TTTCCACACA GATATTATAA CAAAAAATTT CCACCAAACC

Middle_age GCACAGCCGC TTTCCACACA GATATTATAA CAAAAAATTT CCACCAAACC

Middle_age GCACAGCCGC TTTCCACACA GATATTATAA CAAAAAATTT CCACCAAACC

Middle_age GCACAGCCGC TTTCCACACA GATATTATAA CAAAAAATTT CCACCAAACC

Middle_age GTATAGTTGT TTTTTATATA GATATCATAA CAAAAAATTT CCACCAAACC

Middle_age GCACAGCCGC TTTCCACACA GATATTATAA CAAAAAATTT CCACCAAACC

Middle_age GCACAGCCGC TTTCCACACA GATATTATAA CAAAAAATTT CCACCAAACC

Young GCACAGTTGC TTTTTATATA GATATCATAA CAAAAAATTT CCACCAAATT

Young GCACAGCCGC TTTCCACACA GATATTATAA CAAAAAATTT CCACCAAACC

Young GCACAGCCGC TTTCCACACA GATATTATAA CAAAAAATTT CCACCAAACC

Young GCACAGCCGC TTTCCACACA GATATTATAA CAAAAAATTT CCACCAAACC

Young GCACAGCCGC TTTCCACACA GATATTATAA CAAAAAATTT CCACCAAACC

Young GCACAGTTGC TTTTTATATA GATATCATAA CAAAAAATTT CCACCAAATT

Young GCACAGCCGC TTTCCACACA GATATTATAA CAAAAAATTT CCACCAAACC

Young GCACAACCGC TTTCCACACA GATATTATAA CAAAAAATTT CCACCAAACC

Young GCACAGTTGC TTTTTATATA GATATCATAA CAAAAAATTT CCACCAAATT

Young GCACAGCCGC TTTCCACACA GACATCATAA CAAAAAATTT CCACCAAACC

Young GTATAGTTGT TTTTTATATA GATATTATAA CAAAAAATTT CCACCAAACC

143B.TK- GTATAGTTGT TTTTTATATA GATATCATAA CAAAAAATTT CCACCAAACC

HELA GTATAGTTGT TTTTTATATA GATATCATAA CAAAAAATTT CCACCAAACC

Fibroblasts GTATAGTTGT TTTTTATATA GATATCATAA CAAAAAATTT CCACCAAACC

851 900

Untreated CCCCCTCCCC CGCTTCTGGC CACAGCACTT AAACACATCT CTGCCAAACC

Centenarian TCCCTTCCCC CGCTTCTGGC CACAGCACTT AAATATATTT TTGTTAAATT

Centenarian TCCCTTCCCC CGCTTCTGGC CACAGCACTT AAATATATTT TTGTTAAATT

Centenarian TCCCTTCCCC CGCTTCTGGC CACAGCACTT AAATATATTT TTGTTAAATT

Centenarian TCCCTTCCCC CGCTTCTGGC CACAGCACTT AAATATATTT TTGTTAAATT

Centenarian TTTCTTTTCC CGCTTCTGGC CACAGCACTT AAATATATTT TTGTTAAATT

Middle_age CCCCCTCTTT TGCTTCTGGC CACAGCACTT AAATATATTT TTGTTAAATT

Middle_age TTTCTTTTCC CGCTTCTGGC CACAGCACTT AAATATATTT TTGTTAAATT

Middle_age TTTTTTTTCC CGCTTCTGGC CACAGCACTT AAATATATTT TTGTTAAATT

Middle_age CCCCTCTTTT TGCTTCTGGC CACAGCACTT AAATATATTT TTGTTAAATT

Middle_age TTTCTTTTCC CGCTTCTGGC CACAGCACTT AAATATATTT TTGTTAAATT

Middle_age CCCCCTCTTT TGCTTCTGGC CACAGCACTT AAATATATTT TTGTTAAATT

Middle_age TTTCTTTTCC CGCTTCTGGC CACAGCACTT AAATATATTT TTGTTAAATT

Middle_age CCCCCTCTTT TGCTTCTGGC CACAGCACTT AAATATATTT TTGTTAAATT

Middle_age CCCCCTCTTT TGCTTCTGGC CACAGCACTT AAATATATTT TTGTTAAATT

Middle_age CCCCCTCTTT TGCTTCTGGC CACAGCACTT AAATATATTT TTGTTAAATT

Middle_age CCCCTCTTTT TGCTTCTGGC CACAGCACTT AAATATATTT TTGTTAAATT

Middle_age TCCCTTCCCC CGCTTCTGGC CACAGCACTT AAATATATTT TTGTTAAATT

Middle_age CCCCCTCTCT TGCTTCTGGC CACAGCACTT AAATATATTT TTGTTAAATT

Middle_age CCCCCTCTTT TGCTTCTGGC CACAGCACTT AAATATATTT TTGTTAAATT

Young TTTCTTTTCC CGCTTCTGGC CACAGCACTT AAATATATTT TTGTTAAATT

Young CCCCCTCTTT TGCTTCCGGC CACAGCACTT AAATATATTT TTGTTAAATT

Young CCCCCTCTCT TGCTTCTGGC CACAGCACCT AAATATATTT TTGTTAAATT

Young CCCCCTCTTT TGCTTCTGGC CGCAGCACTT AAATATATTT TTGTTAAATT

Young CCCCCTCTTT TGCTTCTGGC CACAGCACTT AAATATATTT TTGTTAAATT

Young TTTCTTTTCC CGCTTCTGGC CACAGCACTT AAATATATTT TTGTTAAATT

Young CCCCCTCTTT TGCTTCTGGC CACAGCACTT AAATATATTT TTGTTAAATT

Young CCCCCTCTTT TGCTTCTGGC CACAGCACTT AAATATATTT TTGTTAAATT

Young TTTCTTTTCC CGCTTCTGGC CACAGCACTT AAATATATTT TTGTTNAATT

Young CCCCTCCCTC CGCTTCTGGC TACAGCACTT AAATATATTT TTGTTAAATT

Young CCCCTTCCCC CGCTTCTGGC CACAGCACTT AAATATATTT TTGTTAAATT

143B.TK- TCCCTTCCCC CGCTTCTGGC CACAGCACTT AAATATATTT TTGTTAAATT

HELA TCCCTTCCCC CGCTTCTGGC CACAGCACTT AAATATATTT TTGTTAAATT

Fibroblasts TCCCTTCCCC CGCTTCTGGC CACAGCACTT AAATATATTT TTGTTAAATT

901 950

Untreated CCAAAAACAA AGAACCCTAA CACCAGCCTA ACCAGATTTC AAATTTTATC

Centenarian TTAAAAACAA AGAACCCTGA CACCAGCCTA ACCAGATTTC AAATTTTATC

Centenarian TTAAAAACAA AGAACCCTGA CACCAGCCTA ACCAGATTTC AAATTTTATC

Centenarian TTAAAAACAA AGAACCCTGA CACCAGCCTA ACCAGATTTC AAATTTTATC

Centenarian TTAAAAACAA AGAACCCTGA CACCAGCCTA ACCAGATTTC AAATTTTATC

Centenarian TTAAAAACAA AGAACCCTAA CACCAGCCTA ACCAGATTTC AAATTTTATC

Middle_age TTAAAAACAA AGAACCCTAA CACCAGCCTA ACCAGATTTC AAATTTTATC

Middle_age TTAAAAACAA AGAACCCTGA CACCAGCCTA ACCAGATTTC AAATTTTATC

Middle_age TTAAAAACAA AGAACCCTAA CACCAGCCTA ACCAGATTTC AAATTTTATC

Middle_age TTAAAAACAA AGAACCCTGA CACCANNCTA ACCNGATTTC AAATTTTATC

Middle_age TTAAAAACAA AGAACCCTGA CACCAGCCTA ACCAGATTTC AAATTTTATC

Middle_age TTAAAAACAA AGAACCCTGA CACCAGCCTA ACCAGATTTC AAATTTTATC

Middle_age TTAAAAACAA AGAACCCTGA CACCAGCCTA ACCAGATTTC AAATTTTATC

Middle_age TTAAAAACAA AGAACCCTAA CACCAGCCTA ACCAGATTTC AAATTTTATC

Middle_age TTAAAAACAA AGAACCCTGA CACCAGCCTA ACCAGATTTC AAATTTTATC

Middle_age TTAAAAACAA AGAACCCTGA CACCAGCCTA ACCAGATTTC AAATTTTATC

Middle_age TTAAAAACAA AGAACCCTAA CACCAGCCTA ACCAGATTTC AAATTTTATC

Middle_age TTAAAAACAA AGAACCCTGA CACCAGCCTA ACCAGATTTC AAATTTTATC

Middle_age TTAAAAATAA AGAATTTTAA TATTAGTTTA ATTAGATTTT AAATTTTATT

Middle_age TTAAAAATAA AGAATTTTAA TATTAGTTTA ATTAGATTTT AAATTTTATT

Young TTAAAAACAA AGAACCCTGA CACCAGCCTA ACCAGATTTC AAATTTTATC

Young TTAAAAATAA AGAACCCTGA CACCAGCCTA ACCNNATTTC AAATTTTATC

Young TTAAAAACAA AGAACCCTGA CACCAGCCTA ACCAGATTTC AAATTTTATC

Young TTAAAAACAA AGAACCCTGA CACCAGCCTA ACCAGATTTC AAATTTTATC

Young TTAAAAACAA AGAACCCTAA CACCAGCCTA ACCAGATTTC AAATTTTATC

Young TTAAAAACAA AGAACCCTGA CACCAGCCTA ACCAGATTTC AAATTTTATC

Young TTAAAAACAA AGAACCCTAA CACCAGCCTA ACCAGATTTC AAATTTTATC

Young TTAAAAACAA AGAACCCTGA CACCAGCCTA ACCAGATTTC AAATTTTATC

Young TTAAAAACAA AGAACCCTAA CACCAGCCTA ACCAGATTTC AAATTTTATC

Young TTAAAAATAA AGAATTTTAA TATTAGTTTA ATTAGATTTT AAATTTTATT

Young TTAAAAACAA AGAACCCTGA CACCAGCCTA ACCAGATTTC AAATTTTATC

143B.TK- TTAAAAATAA AGAATTTTAA TATTAGTTTA ATTAGATTTT AAATTTTATT

HELA TTAAAAACAA AGAACCCTGA CACCAGCCTA ACCAGATTTC AAATTTTATC

Fibroblasts TTAAAANTAA AGAATTTTAA TATTAGTTNA ATTAGATTTT AAATTTTATT

951 1000

Untreated TTTTGGCGGT ATGCACTTTT AACAGTCACC CCCCAACTAA CACATTATTT

Centenarian TTTTGGTGGT ATGCACTTTT AACAGTCATC CCCCAACTAA TACATTATTT

Centenarian TTTTGGTGGT ATGCACTTTT AACAGTCATC CCCCAACTAA TACATTATTT

Centenarian TTTTGGTGGT ATGCACTTTT AACAGTCATC CCCCAACTAA TACATTATTT

Centenarian TTTTGGTGGT ATGCACTTTT AACAGTCATC CCCCAACTAA TACATTATTT

Centenarian TTTTGGCGGT ATGCACTTTT AACAGTCACC CCCCAATTAA CACATTATTT

Middle_age TTTTGGCGGT ATGCACTTTT AACAGTCACC CCCCAATTAA CACATTATTT

Middle_age TTTTGGTGGT ATGCACTTTT AACAGTCATC CCCCAACTAA TACATTATTT

Middle_age TTTTGGCGGT ATGCACTTTT AACAGTCACC CCCCAATTAA CACATTATTT

Middle_age TTTTGGTGGT ATGCACTTTT AACAGTCATC CCCCANCTAA TACATTATTT

Middle_age TTTTGGTGGT ATGCACTTTT AACAGTCATC CCCCAACTAA TACATTATTT

Middle_age TTTTGGTGGT ATGCACTTTT AACAGTCATC CCCCGACTAA TACATTATTT

Middle_age TTTTGGTGGT ATGCACTTTT AACAGTCATC CCCCAACTAA TACGTTATTT

Middle_age TTTTGGTGGT ATGCACTTTT AACAGTCATC CCCCAACTAA TACATTATTT

Middle_age TTTTGGTGGT ATGCACTTTT AACAGTCATC CCCCAACTAA TACATTATTT

Middle_age TTTTGGTGGT ATGCACTTTT AACAGTCATC CCCCAACTAA TACATTATTT

Middle_age TTTTGGCGGT ATGCACTTTT AACAGTCACC CCCCAACTAA CACATTATTT

Middle_age TTTTGGTGGT ATGCACTTTT AACAGTCATC CCCCAACTAA TACGTTATTT

Middle_age TTTTGGTGGT ATGTATTTTT AATAGTTATT TTTTAATTAA TATATTATTT

Middle_age TTTTGGTGGT ATGTATTTTT AATAGTTATT TTTTAATTAA TATATTATTT

Young TTTTGGTGGT ATGCACTTTT AACAGTCATC CCCCAACTAA TACATTATTT

Young TTTTGGTGGT ATGCACTTTT AACAGTCATC CCCCAACTAA TACATTATTT

Young TTTTGGTGGT ATGCACTTTT AACAGTCATC CCCCAACTAA TACATTATTT

Young TTTTGGTGGT ATGCACTTTT AACAGTCATC CCCCAACTAA TACGTTATTT

Young TTTTGGTGGT ATGCACTTTT AACAGTCATC CCCCAACTAA TACATTATTT

Young TTTTGGTGGT ATGCACTTTT AACAGTCATC CCCCAACTAA TACATTATTT

Young TTTTGGCGGT ATGCACTTTT AACAGTCATC CCCCAACTAA TACATTATTT

Young TTTTGGTGGT ATGCACTTTT AACAGTCATC CCCCAACTAA TACATTATTT

Young TTTTGGCGGT ATGCACTTTT AACAGTCACC CCCCAATTAA CACATTATTT

Young TTTTGGTNGT ATGTATTTTT AATAGTTATT TTTTAATTAA TATATTATTT

Young TTTTGGTGGT ATGCACTTTT AACAGTCATC CCCCAACTAA TACGTTATTT

143B.TK- TTTTGGTGGT ACGTATTTTT AATAGTTATT TTTTAATTAA TATATTATTT

HELA TTTTGGTGGT ATGCACTTTT AACAGTCATC CCCCAACTAA TACATTATTT

Fibroblasts TTTNGGTGGN ANGNATTTTT AATAGTTATT TTTTAATTAA TATATTATTT

1001 1050

Untreated TCCCCTCCCA CTCCCATACT ACTAATCTCA TCAATACAAC CCCCGCCCAT

Centenarian TCCCCTCCCA CTCCCATACT ACTAATCTCA TCAATATAAT TTTCGTTTAT

Centenarian TCCCCTCCCA CTCCCATACT ACTAATCTCA TCAATATAAT TTTCGTTTAT

Centenarian TCCCCTCCCA CTCCCATACT ACTAATCTCA TCAATATAAT TTTCGTTTAT

Centenarian TCCCCTCCCA CTCCCATACT ACTAATCTCA TCAATATAAT TTTCGTTTAT

Centenarian TCCCCTCCCA CTCCCATACT ACTAATCTCA TCAATATAAT TTTCGTTTAT

Middle_age TCCCCTCCCA CTCCCATACT ACTAATCTCA TCAATATAAT TTTCGTTTAT

Middle_age TCCCCTCCCA CTCCCATACT ACTAATCTCA TCAATATAAT TTTCGTTTAT

Middle_age TCCCCTCCCA CTCCCATACT ACTAATCTCA TCAATATAAT TTTCGTTTAT

Middle_age TCCCCTCCCA CTCCCATACT ACTAATCTCA TCAATCTAAT TTTCGTTTAT

Middle_age TCCCCTCCCA CTCCCATACT ACTAATCTCA TCAATACAAT CCTTGCTCAT

Middle_age TCCCCTCCCA CTCCCATACT ACTAATCTCA TCAATATAAT TTTCGTTTAT

Middle_age TCCCCTCCCA CTCCCATACT ACTAATCTCA TCAATATAAT TTTCGTTTAT

Middle_age TCCCCTCCCA CTCCCATACT ACTAATCTCA TCAATATAAT TTTCGTTTAT

Middle_age TCCCCTCCCA CTCCCATACT ACTAATCTCA TCAATATAAT TTTCGTTTAT

Middle_age TCCCCTCCCA CTCCCATACT ACTAATCTCA TCAATATAAT TTTCGTTTAT

Middle_age TCCCCTCCCA CTCCCATACT ACTAATCTCA TCAATACAAT CCCCGCCCAT

Middle_age TCCCCTCCCA CTCCCATACT ACTAATCTCA TCAATATAAT TTTCGTTTAT

Middle_age TTTTTTTTAA TTTTAATATA ATAAATTTAA TTAATATAAT TTTAATTTAT

Middle_age TTTTTTTTTA TTTTTATATT ATTAATTTTA TTAATATAAT TTTTGTTTAT

Young TCCCCTCCCA CTCCCATACT ACTAATCTCA TCAATATAAT TTTCGTTTAT

Young TCCCCTCCCA CTCCCATACT ACTAATCTCA TCAATATAAT TTTCGTTTAT

Young TCCCCTCCCA CTCCCATACT ACTAATCTCA TCAATATAAT TTTCGTTTAT

Young TCCCCTCCCA CTCCCATACT ACTAATCTCA TCAATATAAT TTTCGTTTAT

Young TCCCCTCCCA CTCCTATACT ACTAATCTCA TCAATACAAT CCTTGCTCAT

Young TCCCCTCCCA CTCCCATACT ACTAATCTCA TCAATATAAT TTTCGTTTAT

Young TCCCCTCCCA CTCCCATACT ACTAATCTCA TCAATATAAT TTTCGTTTAT

Young TCCCCTCCCA CTCCCATACT ACTAATCTCA TCAATATAAT TTTCGTTTAT

Young TCCCCTCCCA CTCCCATACT ACTAATCTCA TCAATATAAT TTTCGTTTAT

Young TTTTTTTTTA TTTTTATATT ATTAATTTTA TTAATATAAT TTTTGTTTAT

Young TCCCCTCCCA CTCCCATACT ACTAATCTCA TCAATATAAT TTTCGTTTAT

143B.TK- TTTTTTTTTA TTTTTATATT ATTGATTTTA TTAATATAAT TTTTGTTTAT

HELA TCCCCTCCCA CTCCCATACT ACTAATCTCA TCAATATAAT TTTCGTTTAT

Fibroblasts TTTTTTTTTA TTTTTATATT ATTAATTTTA TTAATATAAT TTTTGTTTAT

1051 1100

Untreated CCTACCCAGC ACACACACAC CGCTGCTAAC CCCATACCCC GAACCAACCA

Centenarian TTTACTTAGC ACACACACAC CGCTGCTAAT TTTATATTTT GAATCAACCA

Centenarian TTTACTTAGC ACACACACAC CGCTGCTAAT TTTATATTTT GAATCAACCA

Centenarian TTTACTTAGC ACACACACAC CGCTGCTAAT TTTATATTTT GAATCAACCA

Centenarian TTTACTTAGC ACACACACAC CGCTGCTAAT TTTATATTTT GAATCAACCA

Centenarian TTTACTTAGC ACACACACAC CGCTGCTAAT TTTATATTTT GAATCAACCA

Middle_age TTTTTACAGC ACACACACAC CGCTGCTAAT TTTATATTTT GAATCAACCA

Middle_age TTTACTTAGC ACACACACAC CGCTGCTAAT TTTATATTTT GAATCAACCA

Middle_age TTTACTTAGC ACACACACAC CGCTGCTAAT TTTATATTTT GAATCAACCA

Middle_age TTTACTTAGC ACACACACAC CGCTGCTAAT TTTATATTTT GAATCAACCA

Middle_age CCCTCCCAGC CCATATATAC TGTTGTTAAT TTTATACCTC GAACCAACCA

Middle_age TTTACTTAGC ACACACACAC CGCTGCTAAT TTTATATTTT GAATCAACCA

Middle_age TTTACTTAGC ACACACACAC CGCTGCTAAT TTTATATTTT GAATCAACCA

Middle_age TTTACTTAGC ACACACACAC CGCTGCTAAT TTTATATTTT GAATCAACCA

Middle_age TTTACTTAGC ACACACACAC CGCTGCTAAT TTTATATTTT GAATCAACCA

Middle_age TTTACTTAGC ACACACACAC CGCTGCTAAT TTTATATTTT GAATCAACCA

Middle_age CCTACCCAGC ACACACACAC CGCTGCTAAC CCCATATCCC GAACCAACCA

Middle_age TTTACTTAGC ACACACACAC CGCTGCTAAT TTTATATTTT GAATCAACCA

Middle_age TTAATTTATT ATAAATATTT GGTTGTTAAT TTTATATTTT GAATTAATTA

Middle_age TTTATTTAGT ATATATATAC TGTTGTTAAT TTTATATTTT GAATTAATTA

Young TTTACTTAGC ACACACACAC CGCTGCTAAT TTTATATTTT GAATCAACTA

Young TTTACTTAGC ACACACACAC CGCTGCTAAT TTTATATTTT GAATCAACCA

Young TTTACTTAGC ACACACACAC CGCTGCTAAT TTTATATTTT GAATCAACCA

Young TTTACTTAGC ACACACACAC CGCTGCTAAT TTTATATTTT GAATCAACCA

Young CCTATCCAGC ACATATATAC TGTTGTTAAT TTTATACCTC GAACCAACCA

Young TTTACTTAGC ACACACACAC CGCTGCTAAT TTTATATTTT GAATCAACCA

Young TTTACTTAGC ACACACACAC CGCTGCTAAT TTTATATTTT GAATCAACCA

Young TTTACTTAGC ACACACACAC CGCTGCTAAT TTTATATTTT GAATCAACTA

Young TTTACTTAGC ACACACACAC CGCTGCTAAT TTTATATTTT GAATCAACCA

Young TTTATTTAGT ATATATATAT TGTTGTTAAT TTTATATTTT GAATTAATTA

Young TTTACTTAGC ACACACACAC CGCTGCTAAT TTTATATTTT GAATCAACCA

143B.TK- TTTATTTAGT ATATATATAT TGTTGTTAAT TTTATATTTT GAATTAATTA

HELA TTTACTTAGC ACACACACAC CGCTGCTAAT TTTATATTTT GAATCAACCA

Fibroblasts TTTATTTAGT ATATATATAT TGTTGTTAAT TTTATATTTT GAATTAATTA

1101 1121

Untreated AACCCCAAAG ACACCCCCCA C

Centenarian AATCCCAAAG ACACCCCTTA C

Centenarian AATCCCAAAG ACACCCCTTA C

Centenarian AATCCCAAAG ACACCCCTTA C

Centenarian AATCCCAAAG ACACCCCTTA C

Centenarian AATCCCAAAG ACGCCCCCTA C

Middle_age AACCCCAAAG ACCGCCCCCA C

Middle_age AATCCCAAAG ACGCCCCTTA C

Middle_age AATCCCAAAG ACGCCCCTTA C

Middle_age AATCCCAAAG ACGCCCCTTA C

Middle_age AACCCCAAAG ACACCCCCCA C

Middle_age AATCCCAAAG ACGCCCCATA C

Middle_age AATCCCAAAG ACGCCCCTTA C

Middle_age AATCCCAAAG ACGCCCCTTA C

Middle_age AATCCCAAAG ACGCCCCTTA C

Middle_age AATCCCAAAG ACGCCCCTTA C

Middle_age AACCCCAAAG ACACCCTCCA C

Middle_age AATCCCAAAG ACGCCCCTTA C

Middle_age AATTTTAAAG ATATTTTTTA T

Middle_age AATTTTAAAG ATATTTTTTA T

Young AATCCCAAAG ACGCCCCTTA C

Young AATCCCAAAG ACGCCCCTTA C

Young AATCCCAAAG ACGCCCCTTA C

Young AATCCCAAAG ACGCCCCTTA C

Young AACCCCAAAG ACACCCCCCA C

Young AATCCCAAAG ACGCCCCTTA C

Young AATCCCAAAG ACGCCCCTTA C

Young AATCCCAAAG ACGCCCCTTA C

Young AATCCCAAAG ACGCCCCTTA C

Young AATTTTAAAG ATATTTTTTA T

Young AATCCCAAAG ACGCCCCTTA C

143B.TK- AATTTTAAAG ATATTTTTTA T

HELA AATCCCAAAG ACGCCCCTTA C

Fibroblasts AATTTTAAAG ATATTTTTTA T

**Supplementary File S1.** Multiple alignment of complete mitochondrial D-loop sequences of human DNA samples from blood and cultured cells. In red, the revised Cambridge reference sequence (GenBank: NC_012920, [http://www.mitomap.org](http://www.mitomap.org/MITOMAP/HumanMitoSeq)) is reported. Methylated cytosine residues are highlighted in yellow.
