# Supplementary material for: The Control Region of Mitochondrial DNA Shows an Unusual CpG and Non-CpG Methylation Pattern
Source: DNA Res. 2013 Jun 26;20(6):537–47. doi: 10.1093/dnares/dst029 (PMC3859322; doi:10.1093/dnares/dst029)
Supplement: Supplementary Data [file supp_dst029_dst029supp_fig2.doc]

1 50

Untreated AGTACATAAA TTTACATAGT ACAACAGTAC ATTTATGTAT ATCGTACATT

Blood AGTATATAAA TTTATATAGT ATAATAGTAT ATTTATGTAT ATTGTATATT

3T3-L1 AGTATATAAA TTTATATAGT ATAATAGTAT ATTTATGTAT ATTGTATATT

51 100

Untreated AAACTATTTT CCCCAAGCAT ATAAGCTAGT ACATTAAATC AATGGTTCAG

Blood AAATTATTTT TTTTAAGTAT ATAAGTTAGT ATATTAAATT AATGGTTTAG

3T3-L1 AAATTATTTT TTTTAAGTAT ATAAGTTAGT ATATTAAATT AATGGTTTAG

101 150

Untreated GTCATAAAAT AATCATCAAC ATAAATCAAT ATATATACCA TGAATATTAT

Blood GTTATAAAAT AATTATTAAT ATAAATTAAT ATATATATTA TGAATATTAT

3T3-L1 GTTATAAAGT AATTATTAAC ATAAATTAAT ATATATATTA TGAATATTAT

151 200

Untreated CTTAAACACA TTAAACTAAT GTTATAAGGA CATATCTGTG TTATCTGACA

Blood TTTAAATATA TTAAATTAAT GTTATAAGGA TATATCTGTG TTATCTGACA

3T3-L1 TTTAAATATA TTAAATTAAT GTTATAAGGA TATATGTGTG ATATTTGATA

201 250

Untreated TACACCATAC AGTCATAAAC TCTTCTCTTC CATATGACTA TCCCCTTCCC

Blood TACACCATAC AGTCATAAAC TCTTCTNTTN CATATNACTA TCCCCTTTCC

3T3-L1 TATATTATAT AGTTATAAAT TTTTTTTTTT TATATGATTA TTTTTTTTTT

251 300

Untreated CATTTGGTCT ATTAATCTAC CATCCTCCGT GAAACCAACA ACCCGCCCAC

Blood CATTTGGTCT ATTAATCTAC CATCCTCCGT GAAACCAACA ACCCGCCCAC

3T3-L1 TATTTGGTTT ATTAATTTAT TATTTTTTGT GAAATTAATA ATTTGTTTAT

301 350

Untreated CAATGCCCCT CTTCTCGCTC CGGGCCCATT AAACTTGGGG GTAGCTAAAC

Blood CAATGCCCCT CTTCTCGCTC CGGGCCCATT AAACTTGGGG GTAGCTAAAC

3T3-L1 TAATGTTTTT TTTTTTGTTT TGGGTTTATT AAATTTGGGG GTAGTTAAAT

351 400

Untreated TGAAACTTTA TCAGACATCT GGTTCTTACT TCAGGGCCAT CAAATGCGTT

Blood TGAAACTTTA TCAGACATCT GGTTCTTACT TCAGGGCCAT CAAATGCGTT

3T3-L1 TGAAATTTTA TTAGATATTT GGTTTTTATT TTAGGGTTAT TAAATGTGTT

401 450

Untreated ATCGCCCATA CGTTCCCCTT AAATAAGACA TCTCGATGGT ATCGGGTCTA

Blood ATCGTTTATA TGTTTTCCTT AAATAAGACA TCTCGATGGT ATCGGGTCTA

3T3-L1 ATTGTTTATA TGTTTTTTTT AAATAAGATA TTTTGATGGT ATTGGGTTTA

451 500

Untreated ATCAGCCCAT GACCAACATA ACTGTGGTGT CATGCATTTG GTATCTTTTT

Blood ATCAGTCCAT GATCAACATA ACTGTGGTGT TATGCATTTG GTATTTTTTT

3T3-L1 ATTAGTTTAT GATTAATATA ATTGTGGTGT TATGTATTTG GTATTTTTTT

501 550

Untreated ATTTTGGCCT ACTTTCATCA ACATAGCCGT CAAGGCATGA AAGGACAGCA

Blood ATTTTGGCCT ACTTTCATCA ACATAGCCGT CAAGGCATGA AAGGACAGCA

3T3-L1 ATTTTGGCCT ACTTTCATCA ACATAGCCGT CAAGGCATGA AAGGACAGTA

551 600

Untreated CACAGTCTAG ACGCACCTAC GGTGAAGAAT CATTAGTCCG CAAAACCCAA

Blood CACAGTCTAG ACGCACCTAC GGTGAAGAAT CATTAGTCCG CAAAACCTAA

3T3-L1 TATAGTCTAG ACGCATTTAT GGTGAAGAAT TATTAGTTTG TAAAATTTAA

601 650

Untreated TCACCTAAGG CTAATTATTC ATGCTTGTTA GACATAAATG CTACTCAATA

Blood TTATTTAAGG TTAATTATTC ATGCTTGTTA GATATAAATG CTACTCAATA

3T3-L1 TTATTTAAGG TTAATTATTT ATGCTTGTTA GACATAAATG CTACTCAATA

651 700

Untreated CCAAATTTTA ACTCTCCAAA CCCCCCACCC CCTCCTCTTA ATGCCAAACC

Blood CTAAATTTTA ACTCTCCAAA CCCCCCACCC TTTTCTCTTA ATGCCAAACC

3T3-L1 CCAAATTTTA ACTCTCCAAA CCCCCCACCC CTTCCTCTTA ATGCCAAACC

701 750

Untreated CCAAAAACAC TAAGAACTTG AAAGACATAT AATATTAACT ATCAAACCCT

Blood CCAAAAACAT TAAGAATTTG AAAGATATAT AATATTAATT ATCAAACCCT

3T3-L1 CCAAAAACAT TAAGAATTTG AAAGATATAT AATATTAATT ATTAAATTTT

751 800

Untreated ATGTCCTGAT CAATTCTAGT AGTTCCCAAA ATATGACTTA TATTTTAGTA

Blood ATGTCTCGAT CAATTCTAGT AGTTTTTAAA ATATGACTTA TATTTTAGTA

3T3-L1 ATGTTTTGAT TAATTTTAGT AGTTTTTAAA ATATGATTTA TATTTTAGTA

801 850

Untreated CTTGTAAAAA TTTTACAAAA TCATGTTCCG TGAACCAAAA CTCTAATCAT

Blood CTTGTAAAAA TTCTACAAAA TCATGTTCTG TGAATTAAAA TTTTAATTAT

3T3-L1 TTTGTAAAAA TTTTATAAAA TTATGTTTTG TGAATTAAAA TTTTAATTAT

851 877

Untreated ACTCTATTAC GCAATAAACA TTAACAA

Blood ATTTTATTAT GTAATAAACA TTAACAA

3T3-L1 ATTTTATTAT GTAATAAATA TTAATAA

**Supplementary File S2.** Multiple alignment of complete mitochondrial D-loop sequences of murine DNA samples from blood and cultured cells. In red, the reference sequence (GenBank: NC_005089) is reported. Methylated cytosine residues are highlighted in yellow.
