# Supplementary material for: The Control Region of Mitochondrial DNA Shows an Unusual CpG and Non-CpG Methylation Pattern
Source: DNA Res. 2013 Jun 26;20(6):537–47. doi: 10.1093/dnares/dst029 (PMC3859322; doi:10.1093/dnares/dst029)
Supplement: Supplementary Data [file supp_dst029_dst029supp_fig3.doc]

1 50

untreated AGTACATAAA TTTACATAGT ACAACAGTAC ATTTATGTAT ATCGTACATT

J1 WO AGTATATAAA TTTATATAGT ATAATAGTAT ATTTATGTAT ATTGTATATT

J1 TKO AGTATATAAA TTTATATAGT ATAATAGTAT ATTTATGTAT ATTGTATATT

51 100

untreated AAACTATTTT CCCCAAGCAT ATAAGCTAGT ACATTAAATC AATGGTTCAG

J1 WO AAATTATTTT TTTTAAGTAT ATAAGTTAGT ATATTAAATT AATGGTTTAG

J1 TKO AAATTATTTT TTTTAAGTAT ATAAGTTAGT ATATTAAATT AATGGTTTAG

101 150

untreated GTCATAAAAT AATCATCAAC ATAAATCAAT ATATATACCA TGAATATTAT

J1 WO GTTATAAAAT AATTATTAAT ATAAATTAAT ATATATATTA TGAATATTAT

J1 TKO GTTATAAAAT AATTATTAAT ATAAATTAAT ATATATATTA TGAATATTAT

151 200

untreated CTTAAACACA TTAAACTAAT GTTATAAGGA CATATCTGTG TTATCTGACA

J1 WO TTTAAATATA TTAAATTAAT GTTATAAGGA TATATCTGTG TTATTTGATA

J1 TKO TTTAAATATA TTAAATTAAT GTTATAAGGA TATATCTGTG TTATCTGACA

201 250

untreated TACACCATAC AGTCATAAAC TCTTCTCTTC CATATGACTA TCCCCTTCCC

J1 WO TACACCATAC AGTCATAAAC TCTTCTCTTT CATATGACTA TCCCCTTCCC

J1 TKO TACACCATAC AGTTATNAAC TCTTCTCTTC CATANGATTA TTTTTTTTTT

251 300

untreated CATTTGGTCT ATTAATCTAC CATCCTCCGT GAAACCAACA ACCCGCCCAC

J1 WO CATTTGGTCT ATTAATCTAC CATCCTCCGT GAAACCAACA ATTCGCCCAC

J1 TKO TATTTGGTTT ATTAATCTAC CATCCTCCGT GAAACCAACA ATCCGCCCAC

301 350

untreated CAATGCCCCT CTTCTCGCTC CGGGCCCATT AAACTTGGGG GTAGCTAAAC

J1 WO CAATGCCCCT CTTCTCGCTC CGGGCCCATT AAACTTGGGG GTAGCTAAAT

J1 TKO CAATGCCCCT CTTCTCGCTC CGGGCTCATT AAACTTGGGG GTAGCTAAAC

351 400

untreated TGAAACTTTA TCAGACATCT GGTTCTTACT TCAGGGCCAT CAAATGCGTT

J1 WO TGAAATTTTA TTAGATATTT GGTTTTTACT TCAGGGCCAT CAAATGCGTT

J1 TKO TGAAACTTTA TCAGACATCT GGTTCTTACT TCAGGGCCAT CAAATGCGTT

401 450

untreated ATCGCCCATA CGTTCCCCTT AAATAAGACA TCTCGATGGT ATCGGGTCTA

J1 WO ATCGCCCATG CGTTCCCCTT AAATAAGATA TTTTGATGGT ATCGGGTCTA

J1 TKO ATTGTTTATA TGTTTTTTTT AAATAAGATA TTTTGATGGT ATTGGGTTTA

451 500

untreated ATCAGCCCAT GACCAACATA ACTGTGGTGT CATGCATTTG GTATCTTTTT

J1 WO ATCAGTTTAT GACCAATATA ACTGTGGTGT TATGCATTTG GTATTTTTTT

J1 TKO ATTAGCTCAT GATTAATATA ACTGTGGTGT CATGTATTTG GTATTTTTTT

501 550

untreated ATTTTGGCCT ACTTTCATCA ACATAGCCGT CAAGGCATGA AAGGACAGCA

J1 WO ATTTTGGTTT ATTTTTATTA ATATAGTTGT TAAGGTATGA AAGGATAGTA

J1 TKO ATTTTGGTTT ATTTTTATTA ATATAGTTGT TAAGGTATGA AAGGATAGTA

551 600

untreated CACAGTCTAG ACGCACCTAC GGTGAAGAAT CATTAGTCCG CAAAACCCAA

J1 WO TATAGTTTAG ATGTATTTAT GGTGAAGAAT TATTAGTTTG TAAAATTTAA

J1 TKO TATAGTTTAG ATGTATTTAT GGTGAAGAAT TATTAGTTTG TAAAATTTAA

601 650

untreated TCACCTAAGG CTAATTATTC ATGCTTGTTA GACATAAATG CTACTCAATA

J1 WO TTATTTAAGG TTAATTATTT ATGTTTGTTA GATATAAATG TTATTTAATA

J1 TKO TTATTTAAGG TTAATTATTT ATGTTTGTTA GATATAAATG TTATTTAATA

651 700

untreated CCAAATTTTA ACTCTCCAAA CCCCCCACCC CCTCCTCTTA ATGCCAAACC

J1 WO TTAAATTTTA ATTTTTTAAA TTTTTTATTT TTTTTTTTTA ATGTTAAATT

J1 TKO TTAAATTTTA ATTTTTTAAA GTTTTTATTT TTTTTTTTTA ATGTTAAATT

701 750

untreated CCAAAAACAC TAAGAACTTG AAAGACATAT AATATTAACT ATCAAACCCT

J1 WO TTAAAAATAT TAAGAATTTG AAAGATATAT AATATTAATT ATTAAATTTT

J1 TKO TTAAAAATAT TAAGAATTTG AAAGATATAT AATATTAATT ATTAAATTTT

751 800

untreated ATGTCCTGAT CAATTCTAGT AGTTCCCAAA ATATGACTTA TATTTTAGTA

J1 WO ATGTTTTGAT TAATTTTAGT AGTTTTTAAA ATATGATTTA TATTTTAGTA

J1 TKO ATGTTTTGAT TAATTTTAGT AGTTTTTAAA ATATGATTTA TATTTTAGTA

801 850

untreated CTTGTAAAAA TTTTACAAAA TCATGTTCCG TGAACCAAAA CTCTAATCAT

J1 WO TTTGTAAAAA TTTTATNAAA TTATGTTTTG TGAATTAAAA TTTTAATTAT

J1 TKO TTTGTAAAAA TTTTATAAAA TTATGTTTTG TGAATTAAAA TTTTAATTAT

851 877

untreated ACTCTATTAC GCAATAAACA TTAACAA

**J1 WO** ATTTTATTAT GTAATAAATA TTAATAA

J1 TKO ATTTTATTAT GTAATAAATA TTAATAA

**Supplementary File S3.** Multiple alignment of complete mitochondrial D-loop sequences of DNA samples from wild-type (J1 WT) and triple knockout (J1 TKO) mouse ES cells. In red, the reference sequence (GenBank: NC_005089) is reported. Methylated cytosine residues are highlighted in yellow.
