# Supplementary figures and images for: The Control Region of Mitochondrial DNA Shows an Unusual CpG and Non-CpG Methylation Pattern
Source: DNA Res. 2013 Jun 26;20(6):537–47. doi: 10.1093/dnares/dst029 (PMC3859322; doi:10.1093/dnares/dst029)

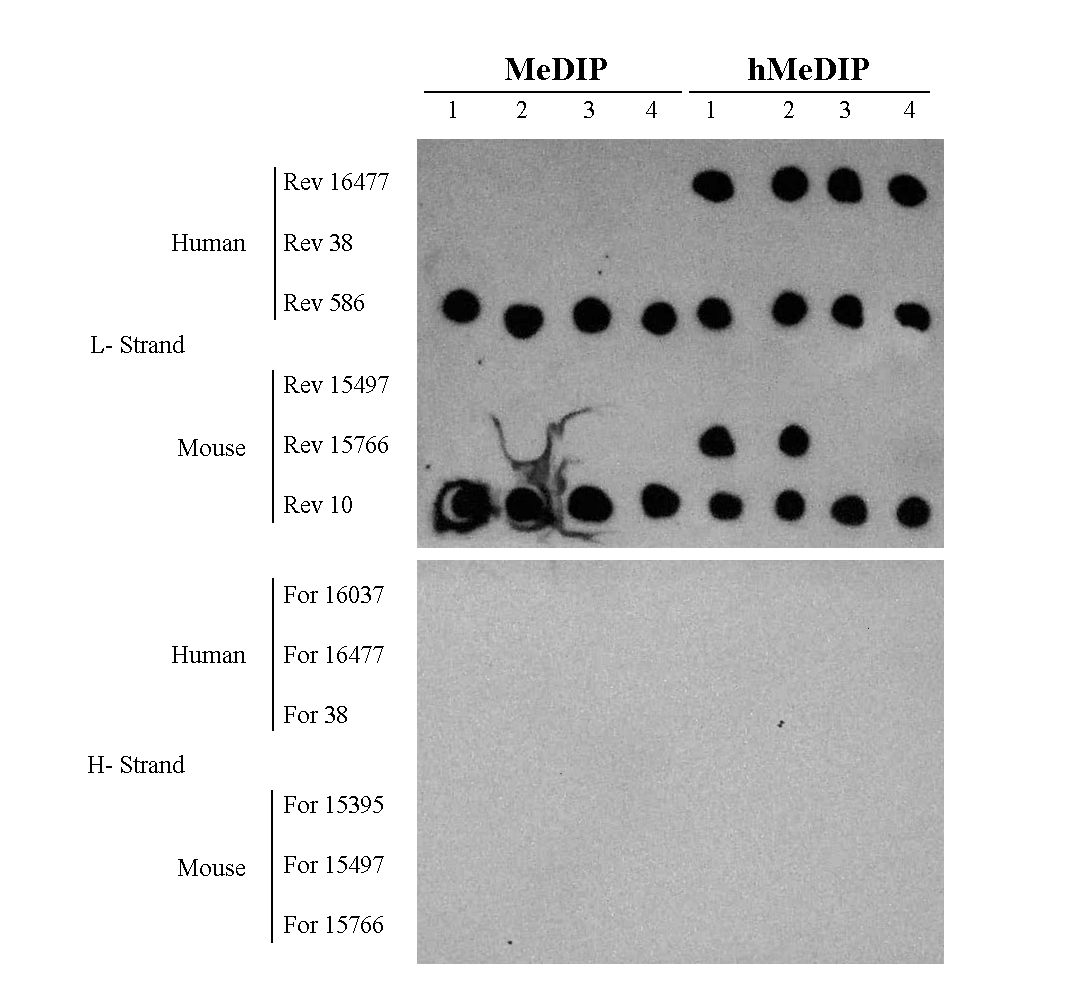

Supplement: Supplementary Data [file supp_dst029_dst029supp_fig4.tif]

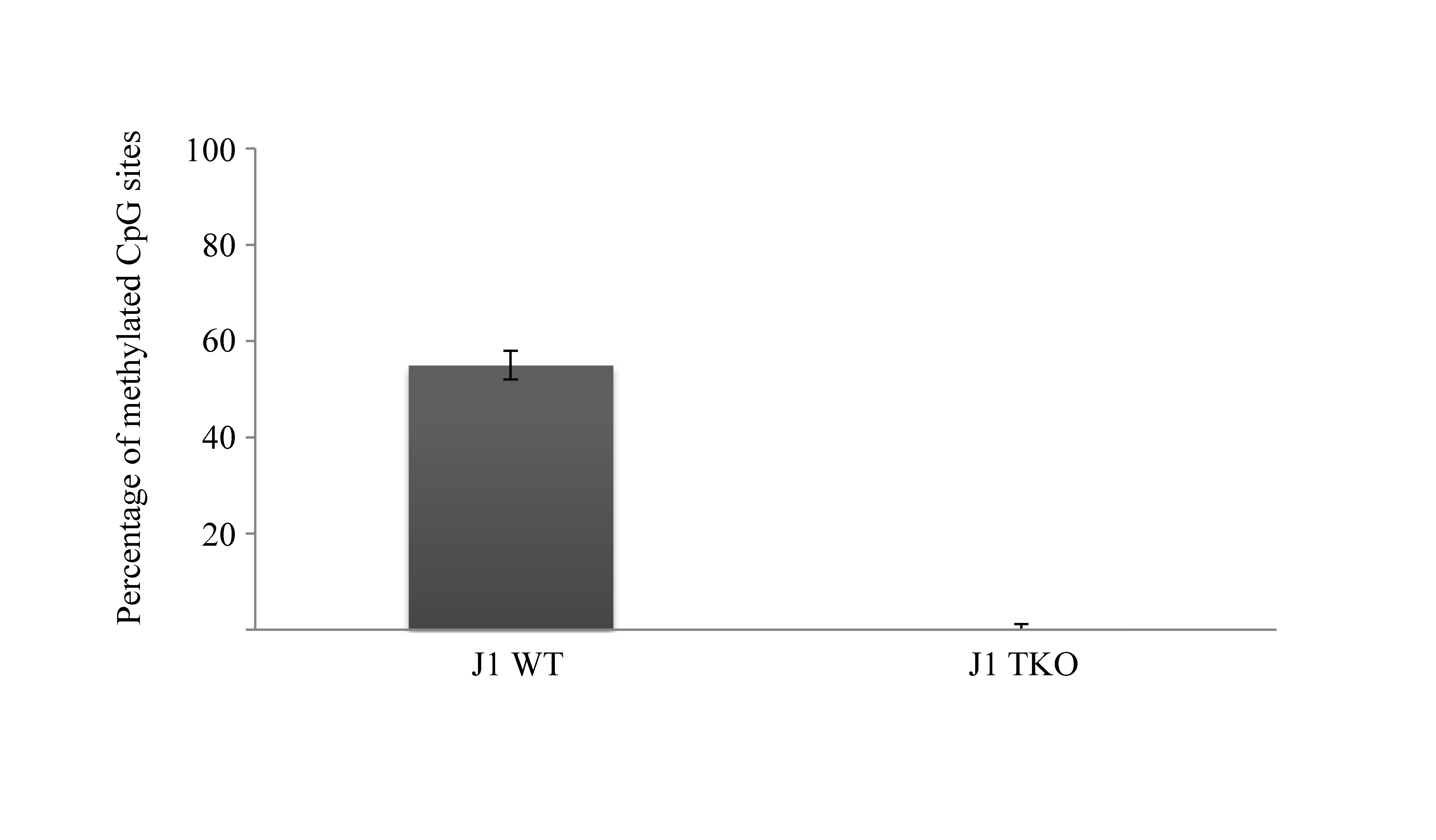

Supplement: Supplementary Data [file supp_dst029_dst029supp_fig5.tif]
