# Supplementary material for: The Control Region of Mitochondrial DNA Shows an Unusual CpG and Non-CpG Methylation Pattern
Source: DNA Res. 2013 Jun 26;20(6):537–47. doi: 10.1093/dnares/dst029 (PMC3859322; doi:10.1093/dnares/dst029)
Supplement: Supplementary Data [file supp_dst029_dst029supp_table1.doc]

**Table S1**. Sequences, localization and annealing temperature of primers amplifying the top (Light) strand used for bisulfite-sequencing procedure. Size of the resulting amplicons is also shown. The 10-bp tags added to primers are indicated in bold.

|  | **Primer name** | **Primer sequence (5'-3')** | | **Primer start position (nt)** | **Annealing**  **temperature (°C)** | **Amplicon size (bp)** |
| --- | --- | --- | --- | --- | --- | --- |
| Human | R1 | For | **AGGAAGAGAG**GATTTTAATTTAAATTATT | 16000 | 42.5 | 215 |
|  |  | Rev | **AGGAAGAGAG**ACTATACTTACTTAT | 16215 |  |  |
|  | R2 | For | **AGGAAGAGAG**ATAAGTAAGTATAGT | 16200 | 44 | 218 |
|  |  | Rev | **AGGAAGAGAG**ATTTCACAAAAAATAATA | 16417 |  |  |
|  | R3 | For | **AGGAAGAGAG**TATTATTTTTTGTGAAATTAA | 16401 | 46 | 151 |
|  |  | Rev | **AGGAAGAGAG**TAAAAAAAACATATAAACTATTT | 16552 |  |  |
|  | R4 | For | **AGGAAGAGAG**AAATAGTTTATATGTTTTTT | 16530 | 44.6 | 212 |
|  |  | Rev | **AGGAAGAGAG**AACATAAATACAATAAATAA | 173 |  |  |
|  | R5 | For | **AGGAAGAGAG**TTATTTATTGTATTTATGTT | 154 | 48 | 202 |
|  |  | Rev | **AGGAAGAGAG**AAAATTTAACAAAAATATA | 356 |  |  |
|  | R6 | For | **AGAGAG**TATATTTTTGTTAAATTTT | 338 | 48 | 290 |
|  |  | Rev | **AGGAAGAGAG**ACCCATCTAAACATTTTCAA | 628 |  |  |
| Mouse | R1 | For | ATGTTTTGATAGTATAAATATTA | 15287 | 48 | 318 |
|  |  | Rev | ATATCCTTATAACATTAATTTAA | 15605 |  |  |
|  | R2 | For | TTAAATTAATGTTATAAGGATAT | 15583 | 46 | 349 |
|  |  | Rev | CCAAAATAAAAAAATACCAAA | 15932 |  |  |
|  | R3 | For | TGGTATTTTTTTATTTTGG | 15911 | 46 | 239 |
|  |  | Rev | TATATCTTTCAAATTCTTAA | 16150 |  |  |
|  | R4 | For | TTAAGAATTTGAAAGATATA | 16144 | 48 | 215 |
|  |  | Rev | AAATACAATTATCCATCTA | 60 |  |  |
