# Supplementary material for: The Control Region of Mitochondrial DNA Shows an Unusual CpG and Non-CpG Methylation Pattern
Source: DNA Res. 2013 Jun 26;20(6):537–47. doi: 10.1093/dnares/dst029 (PMC3859322; doi:10.1093/dnares/dst029)
Supplement: Supplementary Data [file supp_dst029_dst029supp_table2.doc]

**Table S2**. Sequences, localization and annealing temperature of primers amplifying the bottom (Heavy) strand used for bisulfite-sequencing procedure. Size of the resulting amplicons is also shown. The 10-bp tags added to primers are indicated in bold.

|  | **Primer name** | **Primer sequence (5'-3')** | | **Primer start position (nt)** | **Annealing** | **Amplicon size (bp)** |
| --- | --- | --- | --- | --- | --- | --- |
|  |  |  |  |  | **temperature (°C)** |  |
| Human | R1 | For | **AGGAAGAGA**GATTCTAATTTAAACTATT | 16000 | 48 | 214 |
|  |  | Rev | **AGGAAGAGA**GTTGTATTTGTTTGTA | 16214 |  |  |
|  | R2 | For | **AGGAAGAGA**CTTACAAACAAATACAAC | 16197 | 48 | 224 |
|  |  | Rev | TTGATTTTATGGAGGATGGTGG | 16421 |  |  |
|  | R3 | For | CCACCATCCACCATAAAATCAA | 16400 | 48 | 154 |
|  |  | Rev | TTTAAGGGGAATGTGTGGGTTATTT | 16554 |  |  |
|  | R4 | For | AAATAACCCACACATTCCCCTTAAA | 16530 | 48 | 210 |
|  |  | Rev | **GAAGAGAGA**ATGTAGGTGTGATAAATAA | 171 |  |  |
|  | R5 | For | **AGGAAGAGAG**TTATTTATCACACCCTACA | 154 | 48 | 202 |
|  |  | Rev | **GAGA**GGGGGTTTGGTAGAGATGTG | 356 |  |  |
|  | R6 | For | CACATCTCTACCAAACCCC | 338 | 48 | 303 |
|  |  | Rev | TGGGGTGATGTGAGTTTGTT | 641 |  |  |
| Mouse | R1 | For | TATAGAATTATTATATTTGTAAT | 15287 | 46 | 317 |
|  |  | Rev | ATATCCTTATAACATTAATTT | 15604 |  |  |
|  | R2 | For | AATTTGATTATAATATTTTTGT | 15583 | 46 | 349 |
|  |  | Rev | CCAAAATAAAAAAATACCA | 15932 |  |  |
|  | R3 | For | TTATAGAAAAATAAAATTGGATG | 15912 | 46 | 238 |
|  |  | Rev | ATACAAAAAATTCAAAAATCACA | 16150 |  |  |
|  | R4 | For | TGTGATTTTTGAATTTTTTGTAT | 16141 | 46 | 218 |
|  |  | Rev | CCTATATTAATAAATAAATTCAT | 60 |  |  |
