# Supplementary material for: The Control Region of Mitochondrial DNA Shows an Unusual CpG and Non-CpG Methylation Pattern
Source: DNA Res. 2013 Jun 26;20(6):537–47. doi: 10.1093/dnares/dst029 (PMC3859322; doi:10.1093/dnares/dst029)
Supplement: Supplementary Data [file supp_dst029_dst029supp_table3.doc]

**Table S3**. Sequences and localization of primers used in MeDIP/hMeDIP-PCRs. Location within mtDNA of fragments obtained by AluI digestion and size of the resulting amplicons are also shown.

|  | **Primer name** | **Primer sequence (5'-3')** | | **Alu fragment (nt)** | **Primer start position (nt)** | **Amplicon size (bp)** |
| --- | --- | --- | --- | --- | --- | --- |
| Human | M | For | CCTCACCCACTAGGATACCAAC | 16037-16477 | 16261 | 153 |
|  |  | Rev | CACGGAGGATGGTGGTCAAG |  | 16414 |  |
|  | U | For | GGGTACTAAAGCCTAAATAG | 16477-38 | 16516 | 87 |
|  |  | Rev | CGTGAGTGGTTAATAGGG |  | 34 |  |
|  | M | For | ACACCAGCCTAACCAGATTTCAAA | 38-586 | 374 | 171 |
|  |  | Rev | CGGGGTATGGGGTTAGCAG |  | 545 |  |
| Mouse | U | For | GGTATTCTAATTAAACTACTTCT | 15395-15497 | 15398 | 97 |
|  |  | Rev | TATATGCTTGGGGAAAATAG |  | 15485 |  |
|  | U | For | CATACACCATACAGTCATAAA | 15497-15766 | 15621 | 137 |
|  |  | Rev | AAGTTTAATGGCCCGGAG |  | 15758 |  |
|  | M | For | CTTCAGGGCCATCAAATGC | 15766-10 | 15801 | 150 |
|  |  | Rev | GGCTATGTTGATGAAAGTAGG |  | 15950 |  |
